# Alternative Strategy for Development of Dielectric Calcium Copper Titanate-Based Electrolytes for Low-Temperature Solid Oxide Fuel Cells

**DOI:** 10.1007/s40820-024-01523-0

**Published:** 2024-09-26

**Authors:** Sajid Rauf, Muhammad Bilal Hanif, Zuhra Tayyab, Matej Veis, M. A. K. Yousaf Shah, Naveed Mushtaq, Dmitry Medvedev, Yibin Tian, Chen Xia, Martin Motola, Bin Zhu

**Affiliations:** 1 College of Mechatronics and Control Engineering, Shenzhen University, Shenzhen, 518060 People’s Republic of China; 2 Department of Inorganic Chemistry, Faculty of Natural Sciences, Comenius University Bratislava, Ilkovicova, 684215 Bratislava, Slovakia; 3 Energy Storage Joint Research Center, School of Energy and Environment, Southeast University, Nanjing, 210096 People’s Republic of China; 4 Hydrogen Energy Laboratory, Ural Federal University, 620002 Ekaterinburg, Russia; 5 Laboratory of Electrochemical Devices Based on Solid Oxide Proton Electrolytes, Institute of High Temperature Electrochemistry, 620066 Ekaterinburg, Russia; 6 School of Microelectronics, Hubei University, Wuhan, 430062 People’s Republic of China

**Keywords:** LT-SOFCs, Dielectric CaCu_3_Ti_4_O_12_, Semiconductor Ni_0.8_Co_0.15_Al_0.05_LiO_2−*δ*_, Ionic conductivity, Heterostructure electrolyte

## Abstract

The development of low-temperature solid oxide fuel cells (LT-SOFCs) is of significant importance for realizing the widespread application of SOFCs. This has stimulated a substantial materials research effort in developing high oxide-ion conductivity in the electrolyte layer of SOFCs. In this context, for the first time, a dielectric material, CaCu_3_Ti_4_O_12_ (CCTO) is designed for LT-SOFCs electrolyte application in this study. Both individual CCTO and its heterostructure materials with a *p*-type Ni_0.8_Co_0.15_Al_0.05_LiO_2−*δ*_ (NCAL) semiconductor are evaluated as alternative electrolytes in LT-SOFC at 450–550 °C. The single cell with the individual CCTO electrolyte exhibits a power output of approximately 263 mW cm^−2^ and an open-circuit voltage (OCV) of 0.95 V at 550 °C, while the cell with the CCTO–NCAL heterostructure electrolyte capably delivers an improved power output of approximately 605 mW cm^−2^ along with a higher OCV over 1.0 V, which indicates the introduction of high hole-conducting NCAL into the CCTO could enhance the cell performance rather than inducing any potential short-circuiting risk. It is found that these promising outcomes are due to the interplay of the dielectric material, its structure, and overall properties that led to improve electrochemical mechanism in CCTO–NCAL. Furthermore, density functional theory calculations provide the detailed information about the electronic and structural properties of the CCTO and NCAL and their heterostructure CCTO–NCAL. Our study thus provides a new approach for developing new advanced electrolytes for LT-SOFCs.

**Supplementary Information:**

The online version contains supplementary material available at 10.1007/s40820-024-01523-0.

## Highlights

- Dielectric CaCu_3_Ti_4_O_12_ (CCTO) was used as electrolyte in low-temperature solid oxide fuel cells for the first time.
- A new heterostructure electrolyte was designed based on CCTO and Ni_0.8_Co_0.15_Al_0.05_LiO_2−*δ*_ (NCAL). Promising ionic conductivity and high fuel cell performance were achieved
- CCTO–NCAL realized an electrolyte function due to its good dielectric property and a heterojunction effect.

**Supplementary Information:**

The online version contains supplementary material available at 10.1007/s40820-024-01523-0.

## Graphical abstract

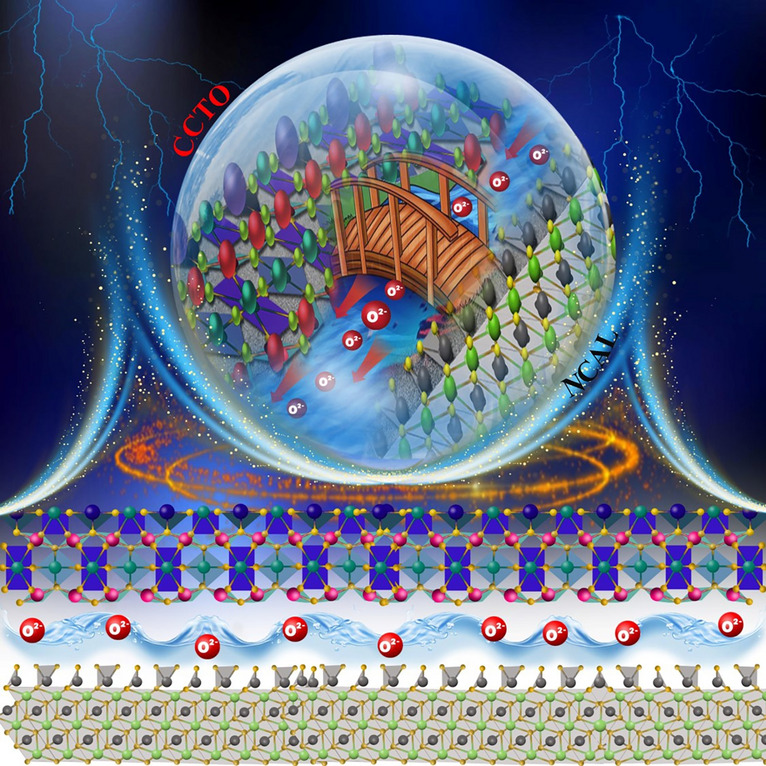

## Introduction

Solid oxide fuel cells (SOFCs) are considered one of the most promising power generation devices for the future, as they have demonstrated the superiority of high energy-conversion efficiency and extremely low pollution [1, 2]. Indeed, there are several limitations associated with SOFCs, such as high costs and technological complexity, because of the high operating temperature condition required by the most commonly used electrolyte, Y_2_O_3_-stabilized ZrO_2_ (YSZ), YSZ requires higher temperature of ~ 1000 °C to motivate its ionic transport to operate fuel cell device [3, 4]. In order to meet these requirements and the growing energy demand, global efforts have been made to develop highly ion-conducting oxides to solve the high temperature bottleneck of SOFCs [2].

A variety of techniques have been attempted to meet the pre-requisites and overcome these bottlenecks [5–7]. For instance, ultrathin film electrolyte layers were developed through micromachined silicon for micro-SOFCs (μSOFCs) to minimize the device ohmic losses and maintain desirable power output at reduced temperatures [8–10]. However, ultrathin films are subjected to severe expensive processes and complex instrumentation. Subsequently, an electrolyte-layer free fuel cell (EFFC) was introduced to eliminate the electrolyte layer, which can be regarded as a significant advancement in fuel cell technology, enabling operation at low temperatures [11, 12]. However, despite the generation of sufficient power output in such systems, the expected practical application of EFFCs has failed to materialize [13]. The controversy surrounding the origin of the increase in conductivity and the suspicion that a high rate of electron/hole transport is unfavorable for electrolytes have hindered the advancement of this technology. Additionally, the question of how short circuiting is prevented without an electrolyte separating layer has not been addressed properly. Consequently, substantial efforts are necessary to elucidate strategies for preventing short circuits and constructing fuel cell devices with novel electrolyte materials to facilitate the advancement and commercialization of fuel cells. Furthermore, fuel cells and other energy conversion and storage devices, including electrostatic capacitors, lithium-ion batteries, and electrochemical capacitors, encompass a diverse range of material applications [14, 15]. Among these materials, various kinds of materials have been reported for LT-SOFCs, including pure ionic conductors [3], semiconductor and their composites [16, 17], and ferroelectric materials [18, 19]. Nevertheless, there is a space available to incorporate dielectric materials into fuel cell technology to determine and realize the intrinsic properties of oxide ions. Dielectric materials have attracted much attention in the field of energy storage devices; specifically, calcium copper titanate CaCu_3_Ti_4_O_12_ (CCTO) was reported for the first time by Subramanian et al. in 2000 [20]. CCTO has excellent potential for use in energy storage applications [21]. Furthermore, CCTO has been reported as a photo-anode for photo-catalysis and photo-electro-catalysis [22]. Compared with other traditional dielectric materials, the environmentally friendly and lead-free non-toxic nature of CCTO makes it an ideal candidate for gas sensors, energy storage, and conversion devices [21, 23]. In addition, CCTO possesses unique physicochemical properties, as reported over other dielectric materials such as ultra-high and stable dielectric permittivity even at high temperatures (20–600 K) [24].

CCTO has been extensively studied from theoretical and experimental perspectives because of its conducting properties [25, 26]. For instance, various models have been investigated to describe grain and subgrain boundary effects [26, 27]. Similarly, Schottky and Poole–Frenkel models were applied to study DC and frequency-dependent electrical properties at high temperatures [28]. Unfortunately, there remains a contradiction in these models, and there is a lack of understanding about charge transport in CCTO, especially with regard to ionic transport behavior. To date, there are no reports on the use of CCTO as an electrolyte. However, the closest such report is that of Braga and Goodenough, who described a dielectric glass electrolyte that can be used to make a safe, low-cost, lithium or sodium ion rechargeable battery with high energy density and long cycle life [29]. Although a limited number of dielectric glass materials have been successfully employed as electrolytes, CCTO was not included in the aforementioned studies [30, 31]. Consequently, there is a significant opportunity to develop CCTO as a more functional material, particularly an O^2−^-conducting electrolyte for advanced LT-SOFCs. In general, CCTO is a wide bandgap (approx. 3.40 eV) *n*-type semiconductor that is electrically heterogeneous and consists of semiconducting grains and insulating grain boundaries [32–34]. Overall, CCTO can be considered a candidate to employ as electrolyte to dig out the intrinsic ionic conducting property. However, its electrical and physical properties at elevated temperatures have never been reported or optimized. This is of particular importance for the use of CCTO as an electrolyte layer in SOFCs.

Inspired by these research gaps, opportunities and challenges, a single-phase cubic perovskite structure based on CCTO was modeled and subsequently constructed. This material was then employed as an electrolyte in fuel cells. The power output of a fuel cell based on the CCTO electrolyte was 263 mW cm^−2^ at 550 °C. This is the first demonstration of using CCTO as an electrolyte in a fuel cell, which exhibited ionic behavior. However, the power output was not efficient at elevated operational temperatures. Therefore, we introduced an appropriate amount of the semiconductor NCAL into dielectric CCTO to prepare *n*-*p* CCTO–NCAL heterojunctions. This heterojunction formation interestingly tunes the dielectric material into a sufficient ionic conductor, consequently suppressing electronic conduction and increasing ionic conduction. Notably, the interface formation in the CCTO–NCAL heterostructure plays a pivotal role in improving and enhancing the ionic conduction. Furthermore, the electrochemical and physical properties of the new CCTO–NCAL heterostructure electrolytes were studied via the use of a range of characterization tools to obtain evidence of the CCTO transition to ionic conducting upon heterostructure formation. The energy band alignment was studied by constructing a heterostructure to tune the energy bands of individual components. Furthermore, density functional theory (DFT) calculations were employed to theoretically study the electronic and structural properties of CCTO, NCAL, and their heterostructure CCTO–NCAL. Additionally, the difference in the charge density of the CCTO/NCAL heterostructure at the (422) and (101) planes was studied.

## Experimental

### Optimization of Crystal Structure and Electronic Properties

The cornerstone of the numerical calculations was performed using DFT as implemented within the Vienna Ab initio Simulation Package (VASP) [35, 36]. Throughout the calculations, the exchange–correlation functional took the Perdew-Burke-Ernzerhof (PBE) form of the Generalized Gradient Approximation (GGA) [37]. To improve the accuracy, pseudo-orbitals were employed using the projector augmented wave (PAW) method [38] and the simplified DFT + U formalism was used for selected potentials [39].

**Fig. 1:**
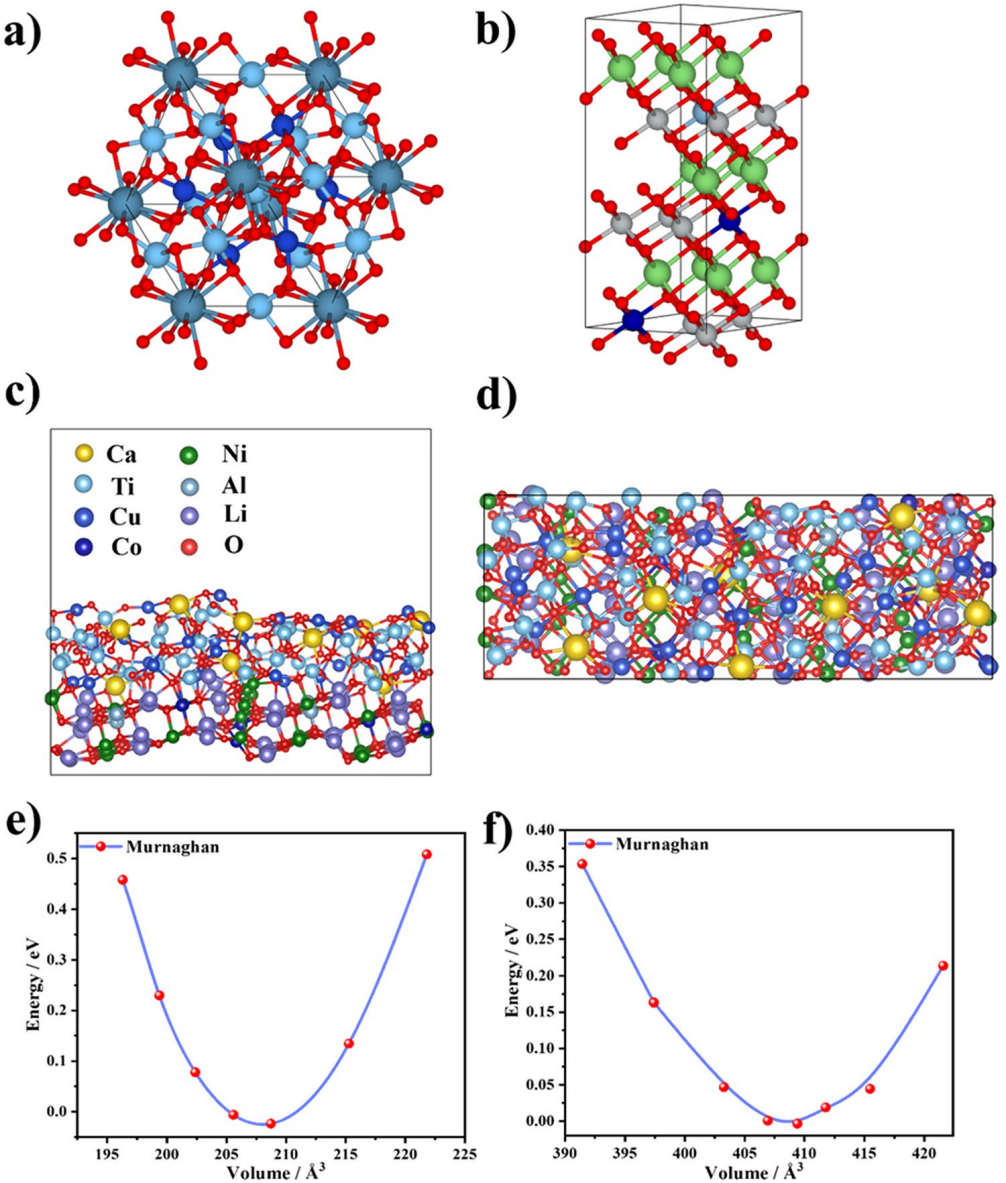
**a**, **b** Optimal and relaxed crystal structures of CCTO and NCAL. **c**, **d** Top and side view of the CCTO–NCAL heterostructure crystal structures and **e**, **f** calculated values of energy vs volume of CCTO and NCAL via Murnaghan method

The reference structures of both NCAL and CCTO were taken from the Crystallography Open Database [39, 40]. The geometry was further optimized using the conjugate-gradient algorithm (CG), where positions as well as cell shape and volume were allowed to vary. The resulting structure was scaled iso-tropically by a small factor and a fixed volume calculation was performed for each such system. The energy surface was interpolated using Murnaghan’s equation of state, and for the volume associated with the minimal energy (Fig. 1e, f) a final calculation was performed where the interatomic forces converged to a threshold of 10^−5^ eV Å^−1^.

The DFT-D3 correction was adopted to describe the weak interactions between atoms [41]. The cutoff energy of the plane-wave basis was set at 450 eV for structural optimization. Brillouin zone integration was performed with 1 × 3 × 1 Monkhorst–Pack k-point sampling to optimize both the geometry and lattice size [42]. A convergence energy threshold of 10^–5^ eV was used for the self-consistent calculations. The equilibrium geometries and lattice constants were optimized with the maximum stress on each atom within 0.02 eV Å^−1^. The spin polarization method was adopted to describe the magnetism of the slab model. The isosurface level of the charge density difference was set at 0.002 eV Å^−3^. The density of states of CCTO(422)/NCALO(101) was obtained using the VASPkit interface [43]. The electronic properties of NCAL and CCTO and the heterostructure are further discussed in Sect. 3.

### Synthesis of Calcium Copper Titanate

The current cubic perovskite-related calcium copper titanate (CaCu_3_Ti_4_O_12_ termed CCTO) material was synthesized by the co-precipitation method. The stoichiometric amounts of calcium nitrate tetra-hydrate Ca(NO_3_)_2_·4H_2_O (Sigma Aldrich 99.99%), copper nitrate tri-hydrate Cu(NO_3_)_2_·3H_2_O (Sigma Aldrich 99.99%), and titanium dioxide (20% in dispersed liquid form, TiO_2_, Sigma Aldrich 99.99%) were used. An appropriate molar ratio of copper and calcium nitrates was used, while commercially purchased 20% TiO_2_ solution in C_2_H_5_OH was dissolved in water to form a stable aqueous solution by a hydrolysis process with continuous stirring and heating (@200 rpm and 80 °C) conditions. Subsequently, the same molar ratio of as-prepared ammonium carbonate (NH₄)₂CO₃ solution was added dropwise into the above solution and was kept stirring until white precipitation. The precipitates were then filtrated and washed several times with absolute ethanol and de-ionized water before drying at 120 °C overnight and grinding in pestle mortal to obtain the powder, followed by sintering at 950 °C for 6 h. The resulting material was thoroughly ground to ensure the homogeneous CCTO powder for characterization as well as fuel cell fabrication.

A commercial semiconductor Ni_0.8_Co_0.15_Al_0.05_LiO_2−*δ*_ (NCAL) was purchased from Tianjin Bamo Sci. & Tech. Joint Stock Ltd., China. Finally, a heterostructure composite of CCTO–NCAL was prepared by mixing CCTO and NCAL in a weight ratio of 7:3 in a similar manner as reported previously [17].

### Preparation of Single-Cell and Fabrication of Fuel Cell Devices

The CCTO–NCAL heterostructure was prepared by mixing an appropriate weight ratio of CCTO and NCAL powders (70:30) via solid-state ball milling for 4 h in alcohol dispersive media, followed by drying in an oven at 120 °C for 6 h and grinding in a mortar and pestle. Subsequently, the composite was sintered at 800 °C for 4 h at a rate of 4 °C min^−1^ in a closed furnace in an air environment and then thoroughly ground for further physical and electrochemical characterization. NCAL has been recently demonstrated as a catalyst capable of triple charge conduction (H^+^/O^2−^/e^−^) and strong catalytic activity in the hydrogen oxidation reaction (HOR) and oxygen reduction reaction (ORR) [44]. The electrodes were prepared by mixing an appropriate amount of NCAL powder into an appropriate volume of terpineol solvent to obtain a slurry, which was then painted/pasted onto Ni foam followed by desiccation at 120 °C to form NCAL-Ni electrodes for the fuel cell device. The fuel cells were fabricated by pressing CCTO and CCTO–NCAL heterostructure powders in between Ni-foam NCAL electrodes on both sides at a pressure of 220 MPa. The thickness of the fuel cell was 1.5 mm, with an active area of 0.64 cm^2^. Silver paste was then applied on both sides of the cells as a current collector. In the same way, the CCTO and CCTO–NCAL cells with Ag electrodes were prepared; the CCTO and CCTO–NCAL pellets were coated with silver electrodes and then sintered at 700 °C (2 °C min^−1^) for 3 h to obtain strong adhesion between the electrodes and pellets before investigating the dielectric properties and electrochemical impedance spectra of the CCTO and CCTO–NCAL heterostructured pellets. All cells were sintered online at 650 °C (2 °C min^−1^) for 3 h prior to operation and measurement.

The cell for gas chromatography–mass spectrometry (GC–MS) characterization was sealed in an Al_2_O_3_ tube using a Pt paste and then Ceramabond 552-VFG sealant (Aremco) and heated to 650 °C.

### Material Characterizations and Electrochemical Measurements

The phase structure of the synthesized pure CCTO, NCAL, and mixed powders was determined using a Bruker D8 advanced X-ray diffractometer (Germany, Bruker Corporation with Cu Kα radiation, *λ* = 1.5418 Å) in a scan range of 2*θ* from 10°–80° with a scanning step of 0.020. MJAD 6.5 software was used for all the refinements. The overall particle morphology, chemical composition, elemental mapping, and qualitative investigation of the size distribution of the synthesized CCTO and its heterostructure with NCAL were studied through scanning electron microscopy (SEM, JEOL JSM7100F Japan) supported by an Oxford energy-dispersive spectrometer (EDS). The internal structure and interface of the as-prepared material were studied through a Tecnai G2 F30 S-TWIN 300 kV/FEG high-resolution transmission electron microscope (HR-TEM). The valence band level maxima were attained via ultraviolet photoelectron spectroscopy (UPS) measurements performed with an unfiltered He-I (21.22 eV) gas discharge lamp and a total instrumental energy resolution of 100 meV. UV‒vis absorption spectra of the materials were obtained with a UV3600 spectrometer (MIOSTECHPTY Ltd.). X-ray photoelectron spectroscopy (XPS) was performed using Al Kα radiation (1486.7 eV) generated from a twin anode X-ray source.

The electric field measurements were carried out to determine the dielectric constant (*ε*′) and dielectric loss (tan *δ*) at frequencies of 20 Hz–3 MHz by an HIKIO IM3590 impedance analyzer at a bias voltage of 0.5 V. Electrochemical impedance spectroscopy (EIS) measurements were carried out by using an electrochemical workstation (Gamry Reference 3000, USA) in open-circuit voltage (OCV) mode. The applied frequency range was 0.1 Hz–1 MHz with an AC signal voltage of 10 mV in amplitude. All measurements were completed in the temperature range of 450–550 °C. ZSIMPWIN software was used to simulate the EIS data based on equivalent circuits. The fuel cell device performance was tested using a DC electronic load instrument (ITECH8511, ITECH Electrical Co., Ltd.). H_2_ and air were used as fuel and oxidant, respectively, with flow rates ranging from 100 to 120 mL min^−1^. The voltage and current were recorded to determine the current density–voltage density (*I*–*V*) and current density-power density (*I*–*P*) characteristics.

The gas product was determined by GC–MS (performed using a SCION GC–MS systems instrument from Bruker Corporation) in temperature conducting detector (TCD) mode, which was employed to analyze the composition of the outlet gas measured at 550 °C. A GC run was repeated every 10 min. The average value of three measurements was taken as the gas volumetric concentration for the Faradaic efficiency calculation, and three average values were used for the plot. The flow rate of H_2_ was 20 mL min^–1^, and open air was used. The gas flow rate was determined using a flowmeter. The Faradaic efficiency was calculated by using Eq. (1):

1 $${\mathrm{FE}}_{{{\mathrm{H}}_{2} {\mathrm{O}}}} = \frac{{0.1315 \times V\left( {{\raise0.7ex\hbox{${{\mathrm{mL}}}$} \!\mathord{\left/ {\vphantom {{{\mathrm{mL}}} {\min }}}\right.\kern-0pt} \!\lower0.7ex\hbox{${\min }$}}} \right) \times v \left( {{\mathrm{mol}}\% } \right)}}{{I_{{{\mathrm{Total}}}} \left( A \right)}} \times 100 \%$$

where $$v \left( {{\mathrm{mol}}\% } \right)$$ is the concentration of H_2_O in the exhaust gas from the electrochemical cell (GC–MS data). $$V\left( {{\raise0.7ex\hbox{${{\mathrm{mL}}}$} \!\mathord{\left/ {\vphantom {{{\mathrm{mL}}} {\min }}}\right.\kern-0pt} \!\lower0.7ex\hbox{${\min }$}}} \right)$$ is the gas flow rate measured using a flow meter at the exit of the electrochemical cell at room temperature and ambient pressure.

## Results and Discussion

First, it is necessary to understand the electronic properties of the as-prepared materials (i.e., CCTO and NCAL) utilizing the DFT calculation to model and construct the optimized and relaxed crystal structures and then considering the relevant density of states, energy band structure, and optical properties. The crystal structures of CCTO and NCAL were optimized considering the (110) plane using VASP with PAW to obtain pure and fine crystal structures and their properties are presented in Fig. 1a, b. The main purpose of this study was to design the structure of CCTO–NCAL heterostructure at the (422) and (101) planes, respectively. The CCTO–NCAL heterostructure was modeled and constructed by considering the layers of CCTO and NCAL through a generalized lattice matching process, as depicted in Fig. 1c, d. The top and side view of the CCTO–NCAL heterostructure are shown in Fig. 1c, d for easy understanding of the optimized crystal structure. Here, the strain at the interface is minimized considering the VASPkit interface. In general, the mean absolute strain should be in the range of 1% to 4% (not more than 5%) to obtain an optimized heterostructure [45, 46]. Moreover, the mean absolute strain of 2.8% was used to obtain the optimized heterostructure in the CCTO (422)/NCAL (101). This indeed confirms the properly constructed CCTO–NCAL heterostructure.

### Procedure for Calculating the Crystal Structures

As a starting point, the reference crystal structure was utilized in Fig. 1a, b. This was optimized utilizing all possible degrees of freedom including not only the cell volume but also the cell shape and individual positions of all atoms. Each iteration involved an explicit calculation of atomic forces, the stress tensor that enabled the aforementioned degrees of freedom to be adjusted, and the whole procedure was repeated until self-consistency was reached. Customary to more precise calculations, the optimized CCTO–NCAL heterostructure was subsequently iso-morphically scaled by a range of small factors (e.g., from 0.99 to 1.03), resulting in a handful of structures that are of slightly different volumes, as depicted in Fig. 1c, d. Each of these structures was further optimized while maintaining the volume constant. Finally, the resulting energy surface is interpolated, and the local minimum of energy versus volume is identified, a procedure referred to as the Murnaghan method (Fig. 1e, f). For the minimum volumes (*V*_o_ CCTO = 208.1 Å^3^, *V*_o_ NCALO = 409.4 Å^3^) the last geometry optimization was optimized (while keeping the volume fixed) using the data from our reference structures.

The optimal parameters after the optimization of the crystal structures are listed in Table 1. The potential energy surfaces of individual bulk structures obtained by DFT depicted in Fig. 1e, f, fitted with Murnaghan curve with effective parameters (*V*_o_, *B*_o_, *B*_o_′) that bear physical significance and describe the equilibrium properties, how strong is the electronic binding, as well as how material is susceptible to pressure, compression and thermal vibrations. Moreover, the curve shows the extent to which the equilibrium geometry has been optimized. For the minimum volumes (*V*_o_ CCTO = 208.1 Å^3^, *V*_o_ NCALO = 409.4 Å^3^) the last geometry optimization was optimized (while keeping the volume fixed) using the data from our reference structures. Moreover, these geometries are further used in the heterostructure calculations where the focus was to put on the interface. Lastly, to double check the validity of those structures, XRD spectra has been calculated solely on these values depicted in Fig. S1. This gives us another means to decipher the experimental lines as well as cross check the values of the mono-structure lines also visible in the powder diffraction of the heterostructure.

**Table 1:** The optimal parameters obtained from the Murnaghan equation of state after optimization of positions, shape, and volume of the unit cell. Values correspond to the local minima of Fig. 1e, f for both CCTO and NCAL

	CCTO	NCAL
*E*_o_	− 148.3 eV	− 276.4 eV
*B*_o_	211.7 GPa	169.2 GPa
*Bo′*	5.5	− 12.9
*V*_o_	208.1 Å^3^	409.4 Å^3^

**Fig. 2:**
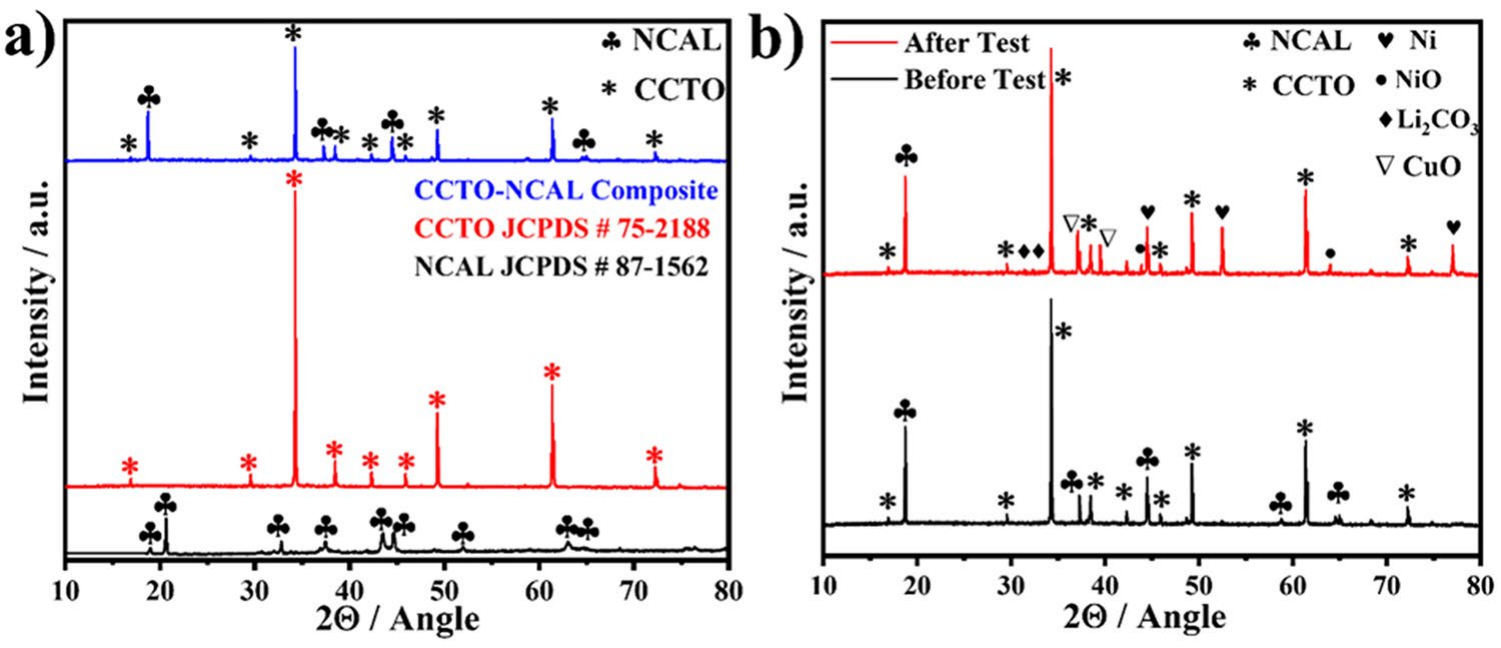
**a** X-ray diffraction patterns of NCAL, CCTO, and their heterostructure CCTO–NCAL. **b** X-ray diffraction patterns of CCTO–NCAL heterostructure before and after testing in fuel cell operation

Initially, theoretical modeling and the construction of the optimal crystal structure of CCTO and NCAL were conducted. Subsequently, the material was prepared experimentally in order to obtain the theoretically calculated structures of the materials, which were then characterized in detail. The XRD pattern of the CCTO, NCAL, and CCTO–NCAL heterostructure displayed in Fig. 2a. For CCTO, a cubic phase perovskite structure was identified via reflection patterns indexed as (110), (211), (220), (130), (222), (231), (400), (422), and (440) planes, respectively, in accordance with the JCPDF file # 75-2188 [47]. The high degree of crystallinity of CCTO is evidenced by sharpness of its diffraction patterns. The XRD pattern of NCAL exhibits a typical layered structure of *α*-NaFeO_2_, as shown in Fig. 2a, which is consistent with the JCPDF file # 87-1562 [48]. To validate the DFT structure optimization of individual CCTO and NCAL, a Rietveld analysis was conducted performed to simulate the XRD pattern with maximum entropy method (MEM) fitting depicted in Fig. S1. A single source (*λ* = 1.54 Å) diffracted from our refined geometry using the RIETAN-FP package was performed [49]. The prominent features are consistent with the experimental results, thereby validating the further use of those structures as a starting point for electronic calculations, as discussed later in the manuscript (e.g., band structures and density of states). The CCTO–NCAL heterostructure showed an integration of the two phases of CCTO and NCAL without the presence of any other diffraction, as depicted in Fig. 2a. Furthermore, the XRD pattern of the CCTO–NCAL as an electrolyte was acquired prior to and after 6 h of testing at 550 °C in contrast to the pure (raw) CCTO–NCAL, as depicted in Fig. 2b. After the online treatment of the CCTO–NCAL electrolyte in H_2_ environment, the formation of CuO, Ni, and NiO diffractions were observed. The formation of NiO is possibly due to the cooling process, during re-oxidation of Ni occurred. This indicates that there were no significant changes in the composition of the materials following the online treatment in the H_2_ environment. It can be seen that the CCTO–NCAL composite, which was sintered at 800 °C for 4 h and subsequently tested online at 550 °C for > 6 h, was thermodynamically stable in terms of structure.

**Fig. 3:**
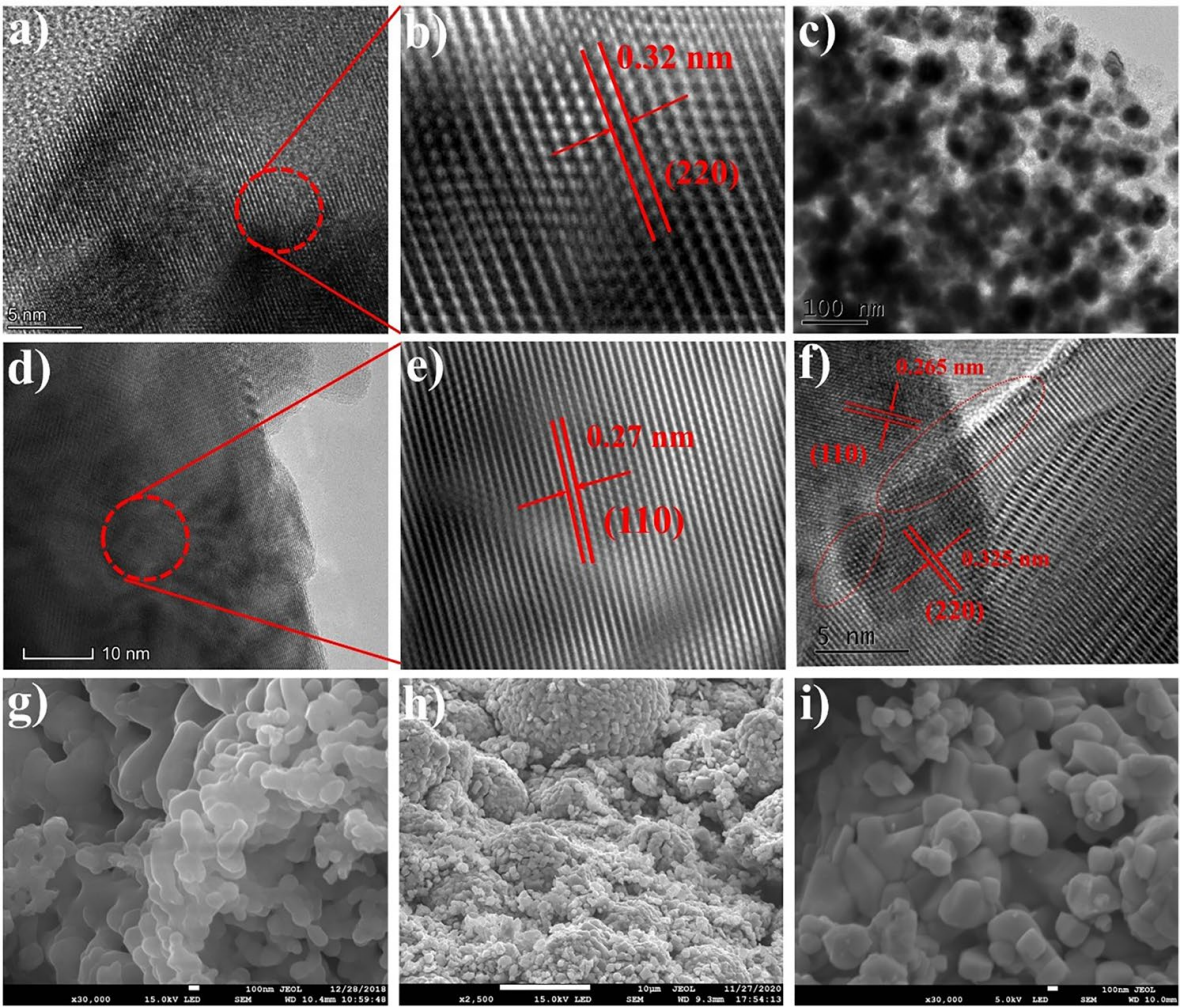
**a**–**f** High-resolution transmission electron microscope images representing the microstructure included the calculated lattice spacing and particle distribution with hetero-interfaces at the particles-level of CCTO, NCAL, and CCTO–NCAL heterostructure. **g**–**i** Field emission-scanning electron microscopy images depicting: the surface morphology and particles distribution of the CCTO, NCAL, and CCTO–NCAL heterostructure

Figure 3a–f shows high-resolution transmission electron microscopy (HR-TEM) images of the microstructures of the CCTO, NCAL, and CCTO–NCAL samples. The crystalline fringes and the corresponding Fourier transform (FFT) patterns of individual CCTO and NCAL and their heterostructures were calculated, as shown in Fig. 3a–f. The CCTO and NCAL particles showed separate, multiple, and even fringes that appeared in the inverted FFT patterns, with calculated d-spacing values of 0.32 and 0.27 nm corresponding to the (220) crystal plane of CCTO and the (110) crystal plane of NCAL, respectively, as shown in Fig. 3a, b and d, e. The heterophasic interfaces between CCTO and NCAL are therefore clearly identified, as annotated in the image depicted in Fig. 3c. Figure 3c was acquired by TEM of CCTO–NCAL at an amplification of 100 nm and shows its homogeneous distribution of nanoscale particles. It is evident that numerous contacts have formed between these particles or grains. In the case of the CCTO–NCAL heterostructure, the interaction of particles of two different phase materials (i.e., CCTO and NCAL) led to an increase in ionic conduction and a suppression of electronic conduction, which assisted in the performance of fuel cells [17]. However, the role of improving ionic conduction and reducing electronic conduction is discussed in detail later in the manuscript. Further insight into the desirable CCTO–NCAL heterostructure at a magnification of 5 nm is shown in Fig. 3f. This displays a nanoscale distribution of particles, confirming the formation of a heterojunction at the interface among particles at the nanoscale in the material. This is important for enhancing ionic transport and fuel cell performance, which will be discussed later. The lattice spacings in CCTO–NCAL were calculated to be 0.325 and 0.265 nm for the (220) and (110) planes of CCTO and NCAL, respectively, as depicted in Fig. 3f. In addition, the wide interface provides a pathway for the transport of ions during electrochemical reactions [50]. Interestingly, a built-in electric field (BIEF) forms at the interface, which supports the fast transport of ionic conduction across the interface [51, 52], as will be discussed later in the manuscript. Figure 3g-i shows the surface morphologies of the as-prepared CCTO, NCAL, and CCTO–NCAL samples obtained by field emission-scanning electron microscopy (FE-SEM). The CCTO particles exhibited an aggregated morphology and had a cubic cylindrical shape but were interconnected (Fig. 3g), while NCAL had a small particle size with irregular shapes and a porous structure, as displayed in Fig. 3h. The formation of the CCTO–NCAL heterostructure at an appropriate weight ratio of 70:30 resulted in ~ 70 and ~ 40 nm particle sizes for CCTO and NCAL, respectively, with good interconnection and adhesion (Fig. 3i). The selected area (electron) diffraction (SAED) pattern of the CCTO–NCAL heterostructure was collected and is shown in Fig. S2.

The elemental distribution of the CCTO–NCAL heterostructure was determined by EDS supported by FE-SEM (Fig. S3a, b). All the elements are roughly distributed according to their compositional ratio. The elemental mapping of CCTO–NCAL is shown in Fig. S3(c–i), where the elemental distributions of Ca, Cu, Ti, Ni, Co, Al, and O are found to be homogeneous.

### Modulating Dielectric Properties for Ionic Conductivity

**Fig. 4:**
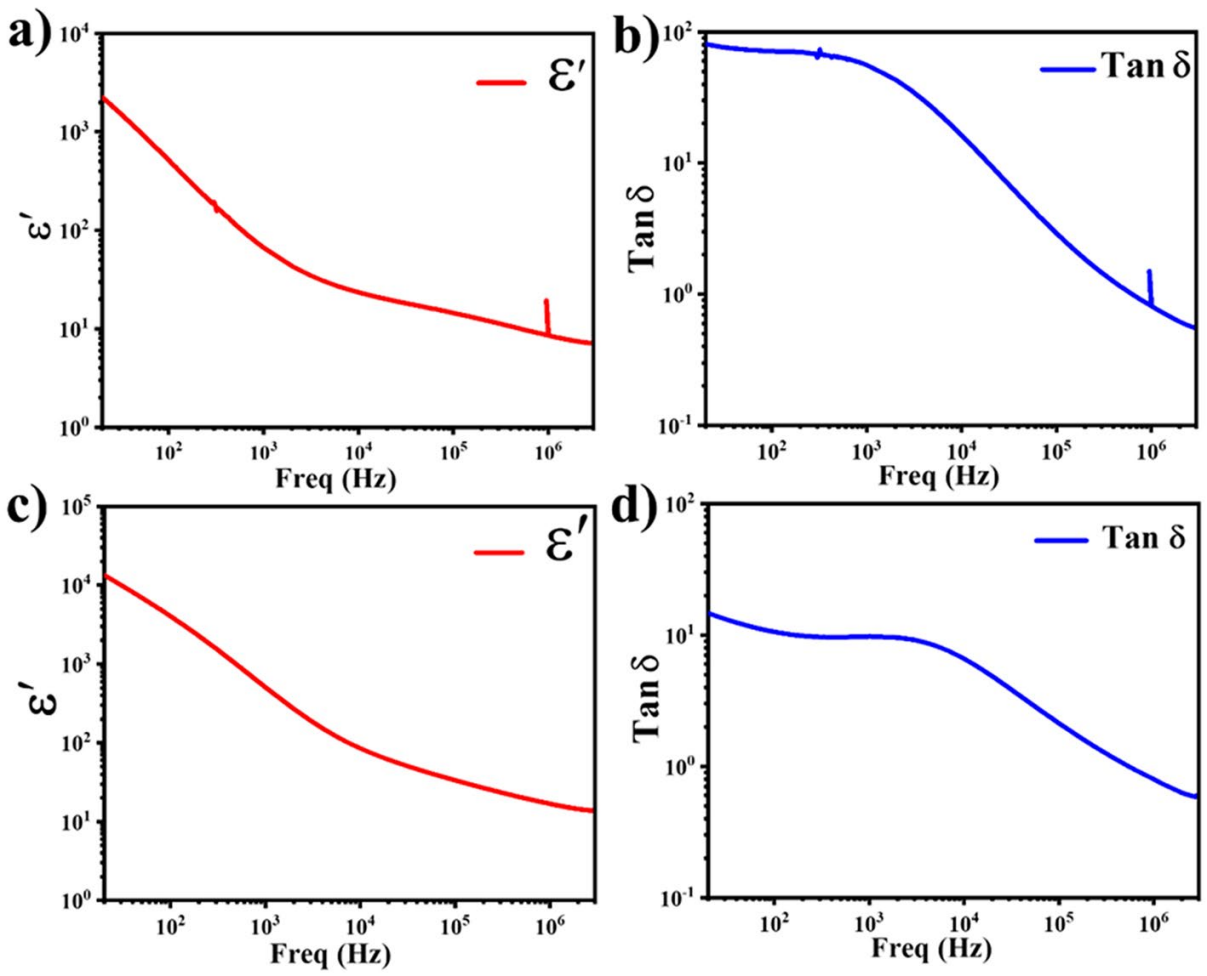
Dielectric constant (*ε*′) and dielectric loss (tan *δ*) of **a, b** CCTO and **c, d** CCTO–NCAL heterostructure samples measured at room temperature in the frequency range of 20 Hz–3 MHz

The effect of synthesis of the heterostructure on the dielectric constant and dielectric loss was the mainly focused in this section of the study of the dielectric characteristics of CCTO and the CCTO–NCAL heterostructure. In this instance, the enhanced functioning of the electrolyte in the fuel cell device is inferred from the dielectric property characterization in response to an increase in the dielectric constant. Yadav et al. reported that a high dielectric constant (*ε*′) facilitates high oxide ion conduction in electrolytes, which is suitable for IT-SOFCs [53]. The dielectric loss (tanδ) and dielectric constant (ε′) real portions of the CCTO and CCTO–NCAL heterostructures at room temperature in the frequency range of 20 Hz–3 MHz are displayed in Fig. 4. In the current case, the ε′ value for CCTO is less than the reported findings; where Table 2 presents a comparison with the previously published dielectric constant of CCTO synthesized at various sintering temperatures. The varying synthesis techniques, testing conditions, and sintering temperatures could be the cause of the low dielectric constant [54]. Furthermore, the values of ε′ for the CCTO–NCAL heterostructure are one order greater than that of pure CCTO, approaching a high value of ~ 10^4.2^ but with a sudden decrease in the dielectric plateau, where tan *δ* is lower than that of pure CCTO, as shown in Fig. 4. The remarkable increase in *ε*′ in the CCTO–NCAL heterostructure may be attributed to the high conductivity of the grain boundaries (GBs), which allows the charge carriers to respond to an electric field to enhance the electric polarization contribution up to higher frequencies [55]. This effect is more dominant, especially when the GB resistance (*R*_gb_) is much lower than the electrode resistance (*R*_e_) due to the addition of NCAL. However, the formation of a dielectric plateau in CCTO is larger than that in CCTO–NCAL, as shown in Fig. 4a, c), which indicates that the dielectric constant of CCTO–NCAL is more effective than that of pure CCTO in different frequency ranges. As a consequence, the increase in ε′ in CCTO–NCAL can play a role to improve the ions conduction due to existence of a dipole moment and also modulate the blockage and streamline the electrons, which is indeed favorable for electrolyte functionality, and the heterointerface holds great potential to help in the fast transport of ionic charge species [56, 57]. Similarly, Nabila et al. has reported a typical dielectric material as a surface-coated BaTiO_3_, where the improved dielectric constant lead to improving the ion’s conductivity in other words ionic conductivity [57]. Consequently, the ions conduction was improved with the increased dielectric constant of surface-coated BaTiO_3_. However, the employment of CCTO–NCAL heterostructure as electrolyte in the fuel cell device (illustrated in the following section of electrochemical characterizations) provided the connection and realization of the above results. Moreover, the electrical properties of epitaxial CCTO, the dielectric constant and the dielectric loss of bulk CCTO measured at 500 and 325 °C have been reported previously, where the dielectric constant was found to increase with increasing temperature along with increasing oxygen ion conductivity in epitaxial CCTO [58, 59]. Thus, the higher dielectric constant in the designed heterostructure composite of a dielectric material with a semiconductor oxide material is a milestone for improving ion conduction and enhancing electrolytic properties.

**Table 2:** Comparison data of dielectric constant of CCTO synthesized at different sintering temperature

**#**	Material used	Sintering temperature (°C)	Dielectric constant	Refs
1	CCTO thin film	1000	~ 10^3.7^	[24]
2	CCTO ceramic	1050	~ 10^6^	[60]
3	CCTO	1100	2.36 × 10^4^	[61]
4	CCTO	950	~ 10^3.3^	Present work

### Electrochemical Characterizations

**Fig. 5:**
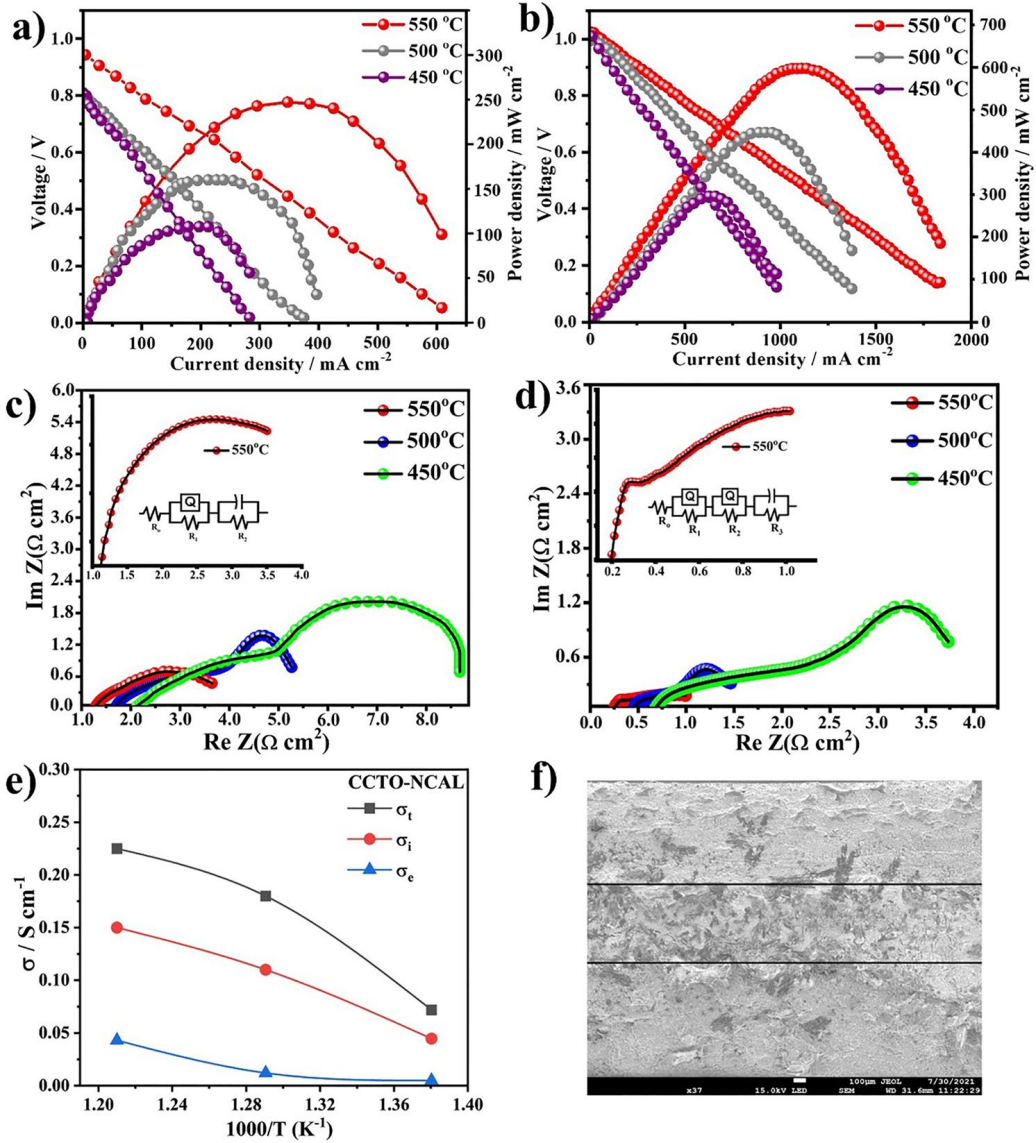
Fuel cell performance in terms of current density and voltage (*I-V*) and current density and power density (*I–P*) of the **a** CCTO and **b** CCTO–NCAL heterostructure electrolyte cells in the H_2_/air environments at operational temperature of 550–450 °C. Impedance spectra of **c** CCTO membrane cell and **d** CCTO–NCAL electrolyte membrane cell at different temperatures and their fitted circuits under H_2_/Air environment. **e** Total, ionic and electronic conductivity contribution in CCTO–NCAL electrolyte membranes in fuel cell conditions at different temperatures. **f** SEM image of cross-sectional view of the fuel cell device with architecture of Ni-NCAL/ CCTO–NCAL /NCAL-Ni cell

Figure 5 presents the typical current density–voltage (*I–V*) and current density-power density (*I–P*) characteristics curves for the CCTO and CCTO–NCAL electrolyte-based fuel cells under H_2_/air operation. The OCV reached 0.95 V for the CCTO electrolyte cell, which delivers a maximum power output of 263 mW cm^−2^ at 550 °C; while the OCV for the CCTO–NCAL heterostructure fuel cell reached 1.02 V along with an improved power density up to 605 mW cm^−2^. Both fuel cells showed promising capability at low-temperature operation. Even down to 450 °C, considerable peak power densities of 95 and 300 mW cm^−2^ were achieved for the CCTO and CCTO–NCAL -based fuel cells, respectively. In Fig. 5a, the high OCV values using the dielectric CCTO itself are of high interest. No serious electronic short-circuiting problem occurred at 550 °C (a minor effect occurred due to 0.95 V OCV). However, significantly improved OCV (> 1.02 V) was observed by introducing the semiconductor phase into the electrolyte layer to form CCTO–NCAL as shown in Fig. 5b. According to traditional fuel cell electrochemistry, the electronic conducting materials are not suitable for use as electrolytes in fuel cells because they lead to severe OCV and performance losses due to electrochemical leakage. In general, CCTO as a dielectric material possesses non-ion conductivity at room temperature while gaining significant oxygen ion conductivity at fuel cell operating temperatures. The promising power outputs and high OCVs refute this claim, as we demonstrate here a promising application of such material system in fuel cells. Furthermore, by introducing the high *p*-type semiconducting material NCAL (9.813 S cm^−1^ at 525 °C) [62–64] into the CCTO to construct a dielectric-semiconductor CCTO–NCAL heterostructure, the fuel cell performance was significantly enhanced as shown in Fig. 5b. The introduction of the semiconductor NCAL has tuned the dielectric properties of the CCTO into a significantly improved oxide-ions conductor at fuel cell operating conditions, and interestingly, the introduction of the semiconducting phase of the NCAL led to the formation of the Schottky junction (SJ) in the CCTO–NCAL heterostructure (discussed later in the manuscript). To show a comparison of power densities and OCV, the histogram of power densities and OCV at different operating temperatures of both CCTO and CCTO–NCAL heterostructure electrolytes in fuel cells is shown in Fig. S4.

For a more detailed investigation of the electrochemical charge transport, EIS spectra were recorded. Figure 5c, d shows the EIS spectra of the CCTO and CCTO–NCAL heterostructure electrolytes operated under H_2_/air at 450–550 °C, respectively. The obtained EIS spectra of the CCTO and CCTO–NCAL electrolytes measured at 550 °C are shown as insets of Fig. 5c, d. Three predominant processes were observed in the EIS spectra of the CCTO electrolyte fuel cell (Fig. 5c). In the case of the CCTO–NCAL electrolyte fuel cell, an additional semicircle was observed at intermediate to low frequencies, as shown in Fig. 5d. A high dielectric constant at low frequencies and a lower polarization resistance in the EIS of CCTO–NCAL were observed for CCTO–NCAL than for CCTO. However, to discuss the EIS simulation, the experimental data of CCTO and CCTO–NCAL were fitted by equivalent circuits *R*_1_-(*QR*_2_)-(*CR*_3_) and *R*_1_-(*QR*_2_)-(*QR*_3_)-(*CR*_4_) using ZSimp-win software, respectively, where R is the resistance, *Q* is the constant phase element, and *C* is the capacitance. The values obtained from EIS of CCTO and CCTO–NCAL in a H_2_/air environment are listed in Table 3. Capacitance is introduced to describe the electrical behavior of mobile carriers that contributes not only to conduction but also to polarization. Based on the data, the charge carriers undergo diffusive motion, e.g., hopping electrons, mobile ions, or even dipoles. The capacitance for each polarization determines the primary mechanism in the fuel cell, either the charge/mass transfer process or the grain/grain boundary. The capacitance for CCTO was approximately 2.83 × 10^–1^ at 550 °C, suggesting a charge/mass transfer process in the fuel cell. An additional polarization resistance was observed in the CCTO–NCAL membrane fuel cell in the intermediate frequency region, which is due to the grain boundary and/or interfacial resistance behavior not observed for CCTO. The CCTO–NCAL heterostructure electrolyte exhibited an overall impedance two orders of magnitude lower than that of CCTO. This decrease in impedance (in terms of resistance at different frequency regions) depicted in Fig. 5c, d and the increase in dielectric constant presented in Fig. 4c, d reflect the enhanced ionic transport and suppressed electronic conduction in CCTO–NCAL. Therefore, the EIS spectra show that the formation of the CCTO–NCAL heterostructure significantly supports charge transport in terms of ion conduction better than that of pure CCTO, as depicted in Fig. 5c, d. The charge transport was further understood by calculating the total electrical conductivity of CCTO and CCTO–NCAL by the following Eq. (2):

2 $$\sigma = {\raise0.7ex\hbox{$L$} \!\mathord{\left/ {\vphantom {L {R \times S}}}\right.\kern-0pt} \!\lower0.7ex\hbox{${R \times S}$}}$$

**Table 3:** EIS fitted data obtain for CCTO and CCTO–NCAL using NCAL an electrodes electrolyte membranes using ZSimpWin software, where n is frequency power, *n* [0 < *n* < 1]

Composition (°C)	*R*_1_ (Ω cm^2^)	*R*_2_ (Ω cm^2^)	*Q*_1_ (CPE_1_) *Y*_o_[(*S* − *s*)^*n*^ cm^−2^]	Q_2_ (CPE_2_) *Y*_o_[(*S* − *s*)^*n*^ cm^−2^]	*R*_3_ (Ω cm^2^)	*R*_4_ (Ω cm^2^)	*C*_1_ (F cm^–2^)	*n*
*CCTO*								
550	1.201	1.412	5.28E−03		0.921		2.83E−01	3.36E−01
500	1.704	2.312	1.63E−02		1.411		1.13E−01	1.66E−01
450	2.101	2.842	6.90E−03		4.845		5.25E−07	2.10E−01
CCTO–NCAL								
550	0.201	0.212	5.97E–4	0.1251	0.66	–	1.12E−05	1.12E−01
500	0.414	0.505	2.887	0.2355	0.08	0.4	1.04E−05	6.73E–01
450	0.612	0.388	0.05023	0.009546	1.402	1.25	0.1647	1.26E−01

(where *L* shows the thickness of the electrolyte membrane layer, and *S* is the effective area). In addition, the ionic conductivity of CCTO–NCAL and CCTO was studied by an unconventional method [17], where Ohm’s law was used to calculate the values of the ionic conductivity utilizing the respective obtained *I–V* polarization curves (as shown in Fig. 5a, b). This is reliable, as the linear part of the polarization curve at low-intermediate current region represents the total ohmic polarization loss (Δ*V*_ohm_) of the tested fuel cell device, which is triggered by the Ohmic resistances of electrolyte and electrodes [65]. In this study, the total Ohmic resistance obtained from the polarization curve was considered similar to the ionic resistance of the CCTO–NCAL electrolyte. Indeed, the electronic resistance of electrodes (NCAL/oxidized Ni-foam) is negligible in contrast to the ionic resistance of the CCTO–NCAL electrolyte [65, 66]. Thus, the area-specific resistance (*R*_ASR_) of the CCTO–NCAL electrolyte was expressed by Eq. (3) as:

3 $${\mathrm{ASR}} = {\raise0.7ex\hbox{${\Delta V_{{{\mathrm{ohm}}}} }$} \!\mathord{\left/ {\vphantom {{\Delta V_{{{\mathrm{ohm}}}} } {\Delta I_{{{\mathrm{ohm}}}} }}}\right.\kern-0pt} \!\lower0.7ex\hbox{${\Delta I_{{{\mathrm{ohm}}}} }$}}$$

in terms of Δ*V*_ohm_ and the corresponding current drop (Δ*I*_ohm_) [67], which is the slope of the *I-V* characteristic curve at the Ohmic polarization part. By this, the ionic conductivity (*σ*_*i*_) of CCTO–NCAL and CCTO electrolytes was estimated according to the following Eq. (4) based on the *I–V* curves:

4 $$\sigma_{i} = {\raise0.7ex\hbox{$L$} \!\mathord{\left/ {\vphantom {L {R_{{{\mathrm{ASR}}}} \times S}}}\right.\kern-0pt} \!\lower0.7ex\hbox{${R_{{{\mathrm{ASR}}}} \times S}$}} = {\raise0.7ex\hbox{${\Delta I_{{{\mathrm{ohm}}}} \times L}$} \!\mathord{\left/ {\vphantom {{\Delta I_{{{\mathrm{ohm}}}} \times L} {\Delta V_{{{\mathrm{ohm}}}} \times S}}}\right.\kern-0pt} \!\lower0.7ex\hbox{${\Delta V_{{{\mathrm{ohm}}}} \times S}$}}$$

**Fig. 6:**
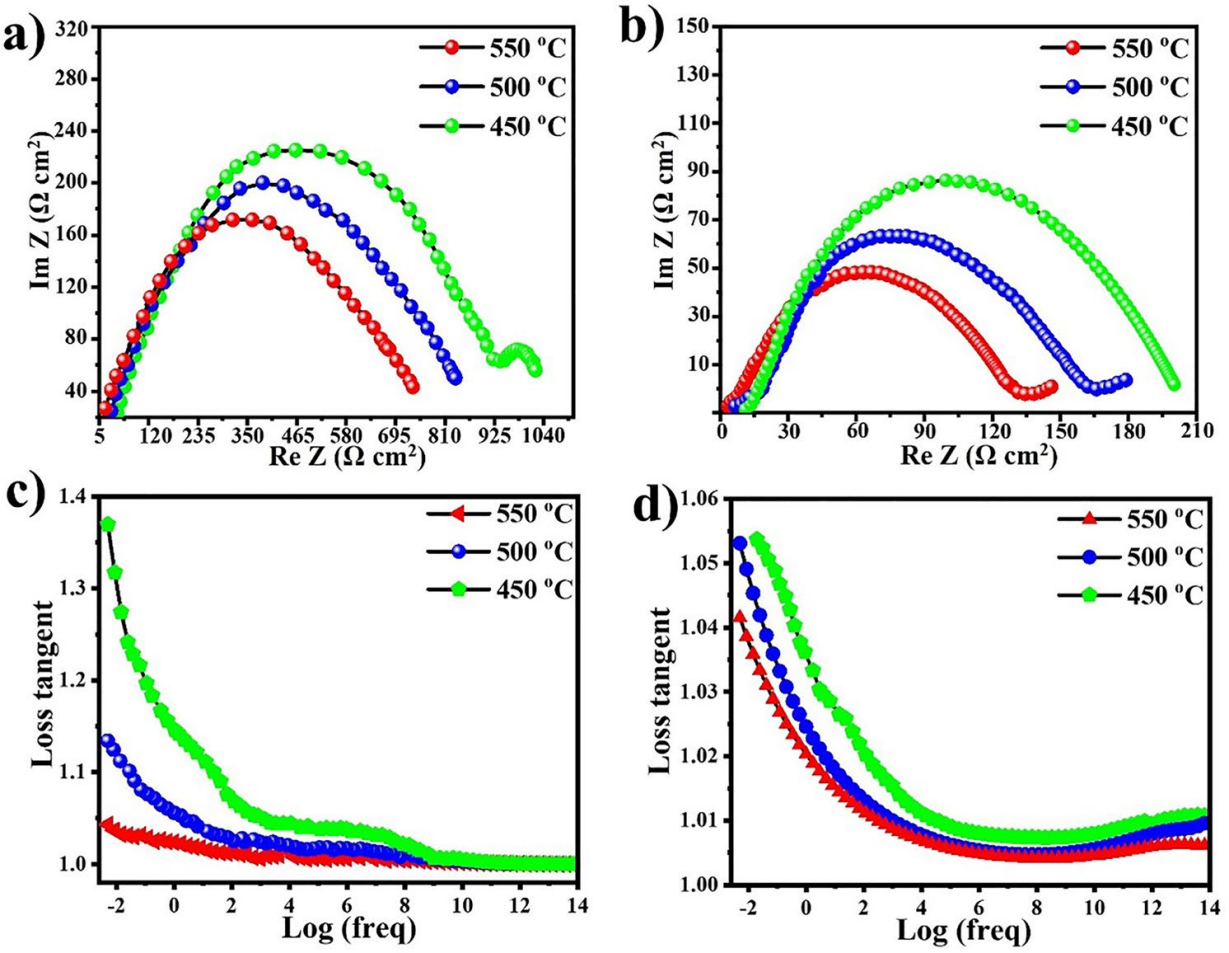
Impedance spectra of **a** CCTO and **b** CCTO–NCAL using Ag electrodes; frequency dependence of loss tangent (tan *δ*) for **c** CCTO and **d** CCTO–NCAL membrane at selected temperatures

The electronic conductivity was calculated by subtracting the ionic conductivity from the total conductivity. A similar procedure was used to calculate the conductivities of CCTO. The calculated electronic and ionic conductivities of CCTO are 0.08 and 0.098 S cm^−1^, respectively, at 550 °C, while for CCTO–NCAL heterostructure the electronic conductivity decreased to 0.041 S cm^−1^ (with the initial value of 0.08 S cm^−1^). The ionic conductivity was increased from 0.098 to 0.15 S cm^−1^, as shown in Figs. 5e and S5. As observed, the electronic conduction was suppressed in the CCTO–NCAL electrolyte, and the ionic conductivity was significantly enhanced by the formation of the CCTO–NCAL heterojunction. The total conductivity of CCTO obtained from the EIS spectra in air shows a poor value of 2.2 × 10^–3^ S cm^−1^ at 550 °C, but it improved significantly to 1.01 × 10^–2^ S cm^−1^ for CCTO–NCAL heterostructure as shown in Fig. S6 (obtained from the EIS performed in air (Fig. 6a, b). The formation of CCTO–NCAL heterostructure tuned the conductivity and promising conductivity was achieved in both cases. On the other hand, the annealing environment showed an effect on the overall electrical conductivity, i.e., CCTO annealed in air contained lower grain boundary resistance compared to those annealed in other environments (in this study, annealing was performed in air).

In addition, the fuel cell cross-sectional distribution of the cell with electrolyte and electrodes is displayed in Fig. 5f, which confirms the successful construction of the fuel cell device and illustrates the morphology of the electrolyte and electrodes as well as the formation of the electrolyte and electrode interface. The electrolyte has a tight structure after high-pressure pressing and online sintering, which at least does not allow gases diffusion, which is appropriate for the fuel cell device. The formed CCTO–NCAL interface plays a crucial role in improving the overall ionic conductivity, where there is a formation of built-in electric field at the interface that accelerate and channelizes the ions transport [51, 52]. The formation of the interface without decay leads to high charge transport of ions and supports the triple-phase boundary process at the electrolyte/electrode interface. All these factors resulted in the remarkable performance of the fuel cell device. A question is raised regarding how the CCTO–NCAL heterostructure with semiconductor phase avoids electronic short-circuiting problems while achieving enhanced ionic conductivity. This is indeed unusual from conventional SOFC science. The mechanism is discussed below from a dielectric point of view in detail.

In addition, SEM cross-sectional view images of the fuel cell device were taken before and after cell operation (Fig. S7a, b). A small crack was observed near the electrolyte and electrode in the lower portion of cross-section, possibly due to the stress of the operating tool or crushing damage during the scissoring of the fuel cell pellet for SEM characterization (see Fig. S7a). The surface morphology of the cross-section of the fuel cell devices did not change and showed no large cracks. In fact, a tighter morphology can be observed in Fig. S7b to prove its low porosity and suitable tightness to stop the H_2_-air cross-over across the electrolyte. In addition, the cross-sectional SEM image of the fuel cell device, including the electrolyte and the electrode, was detected to show the fine interface formation. It illustrates that the interface formation without any cracks that highly support a large number of triple phase boundary’s reaction sites (displayed in Fig. S7c). Furthermore, the internal morphology of the electrolyte is almost similar after testing the cell, which indicates that no loss of density contributes to the gas tightness and avoids gas leakage (see Fig. S7d, e). The presence of carbonates also plays a crucial role in the high gas tightness of electrolyte because sodium carbonate should be molten into liquid phase during the cell operation at fuel cell operational temperature, the molten carbonate filled in the support pores between particles and no open pores are visible on the membrane. However, the distribution of particles and the formation of a large number of interfaces in the as-prepared CCTO–NCAL heterostructure may result in more reaction sites than a single CCTO, and consequently improve the ionic transport in the composite material through the interfacial conduction, which directly benefits the performance of fuel cell [68]. Therefore, it can be observed that the electrolyte membrane has good porosity, and interestingly, the larger thermal expansion co-efficient of NCAL 11.1 × 10^–6^ K^−1^ than CCTO (9.9 × 10^–6^ K^−1^) obtained in temperature range from 300–1000 K can reasonably fill some residual porosity at certain operational temperatures resulting to a gas-tight electrolyte causing good OCVs. In addition, the morphology of the NCAL-Ni electrode is also provided to illustrate the porous morphology of the electrode (see Fig. S7e) and a clear surface morphology of the electrode can be observed.

### Joint Correlation of Dielectric Properties and Ionic Transport

To specify the improved OCV and enhanced power output in CCTO–NCAL heterostructure electrolyte-based fuel cells, dielectric response to frequency and electrochemical analysis were carried out to understand their inter-relationship. For this purpose, the real part of the dielectric constant (*ε*_*r*_), dielectric loss, and EIS spectra were considered and the data are presented in Figs. 4 and 6. The frequency dependence *ε*_*r*_ values in low-frequency region anomalously reached > 10^4.2^ at 100 Hz depicted in Fig. 4c. High *ε*_*r*_ values at lower frequencies are due to the superimposed interfacial polarization of oxygen vacancies associated with the interface formation between CCTO and NCAL in the heterostructure. Thus, high *ε*_*r*_ of CCTO–NCAL was generated due to the interfacial polarization, which assisted in stopping the intrinsic and generated electrons. In CCTO, there are intrinsic dipoles, ionic and electronic polarization contributions, whereas the interfacial contribution between CCTO and electrode is regarded as an extrinsic effect due to its dielectric loss, which has less influence on the ions transport inside the CCTO membrane. However, considering the EIS spectrum of CCTO–NCAL, the interfacial polarization is derived from the charge transfer of electrons and ions between grain boundaries and bulk electrode interfaces, which leads to an enormous apparent dielectric constant of CCTO–NCAL, ranging from 10^3.3^ and 10^4.2^ corresponds to CCTO and CCTO–NCAL presented in Fig. 4a, c. Thus, by introducing NCAL into CCTO, higher values of *ε*_*r*_ lead to reduced electron leakage in terms of high OCVs and enhancement of ionic transport as detected in the CCTO–NCAL electrolyte layer [53]. An increase in *ε*_*r*_ has a direct effect on the OCV due to the blockage effect and streamline of electrons and promotion of oxide ions, while the impedance was reduced by two times in the case of the CCTO–NCAL heterostructure membrane using different electrodes, such as NCAL-Ni and Ag, operated under H_2_/air and air environments compared to that of pure CCTO with NCAL-Ni and Ag electrodes under H_2_/air and air environments, as depicted in Figs. 5a–d and 6a–d, respectively. Quantitative analysis of these polarizations provided an understanding of the relationship between the apparent dielectric properties and polarization behaviors of CCTO–NCAL. Hence, the dielectric spectrum from low to high frequencies quantifies all the polarization contributions that were continuously measured.

To obtain further insight, EIS spectra were recorded. Figure 6a, b shows the EIS spectra of CCTO and the CCTO–NCAL heterostructure with Ag paste electrodes operated under an air atmosphere at temperatures ranging from 450 to 550 °C, respectively. To gain further insight into the underlying processes, the tangent loss (tan *δ*) values obtained from the EIS of the CCTO and CCTO–NCAL with Ag electrodes in an air atmosphere as a function of frequency at various temperatures (depicted in Fig. 6a–d) were analyzed. The data extracted from the EIS spectra of the CCTO and CCTO–NCAL with Ag electrodes in an air environment are listed in Table 4. The obtained tan *δ* values are compared with the tan *δ* relaxation process displayed in Fig. 4b, d. In both cases, the relaxation pattern and the polarization are in phase with the electric field frequency at low frequencies; however, at higher frequencies, the polarization lags behind the electric field frequency in CCTO and CCTO–NCAL. The mobile charges (contained in the electrolyte layer) are not susceptible to following the higher frequency electric field in CCTO and CCTO–NCAL. The tan *δ* peaks observed in Fig. 6c, d displayed greater widths for the CCTO peak than for the CCTO–NCAL peak. This indicates that the relaxation time of the ion species in CCTO is longer than that in CCTO–NCAL, which provides more pathways for ion transport in CCTO–NCAL. The shift in peaks to higher frequencies indicates an increase in conductivity, as measured by EIS at different temperatures in air.

**Table 4:** EIS fitted data obtain for CCTO and CCTO–NCAL electrolyte membranes with Ag electrodes in air using ZSimpWin software, where n is frequency power, *n* [0 < *n* < 1]

Composition (°C)	*R*_1_ (Ω cm^2^)	*R*_2_ (Ω cm^2^)	*Q* (CPE) *Y*_o_[(*S* − *s*)^*n*^ cm^−2^]	*R*_3_ (Ω cm^2^)	*C*_1_ (F cm^–2^)	*n*
*CCTO*						
550	21.54	15.67	4.196E−8	659.79	1.302E−10	0.666
500	35.87	24.52	1.691E−7	778.11	1.318E−10	0.5856
450	48.45	876.55	1.243E−9	115.87	3.18E−6	0.8324
*CCTO–NCAL*						
550	5.23	104.77	2.228E−4	43.52	2.597E−9	0.8
500	7.042	160.96	1.287E−4	25	1.44E−8	0.7
450	11.42	180.48	1.131E−4	8.42	5.723E−8	0.334

**Fig. 7:**
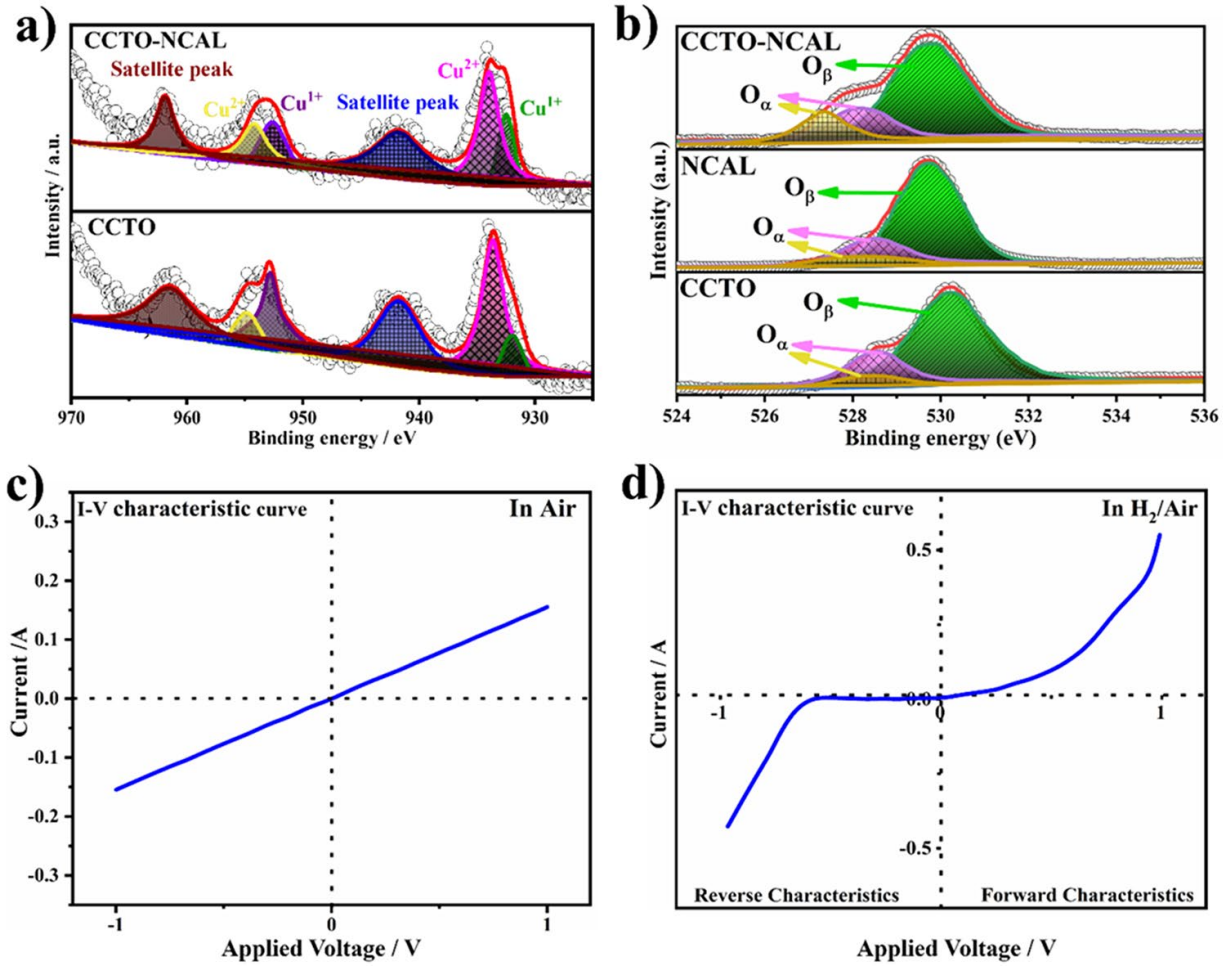
XPS spectra of **a** Cu-2*p* spectra of CCTO and CCTO–NCAL. **b** O 1*s* spectra of CCTO, NCAL and CCTO–NCAL. **c, d** Device *I-V* characteristics curves of the CCTO–NCAL composition-based Schottky junction fuel cell devices measured under various bias voltages under different environments of air and H_2_/air

The introduction of dielectric materials (CCTO) into the electrolyte layer represents a novel approach in the field of LT-SOFCs. Consequently, the defect structure of CCTO was considered to gain insight into ionic conduction and facilitate the tuning of dielectric materials into fuel cell electrolytes. Accordingly, the structural defect model postulates that there is a smaller distance between Ca atoms in their adjacent unit cells. It is believed that the distance to be supported by oxygen vacancies, which are formed as a result of the reduction of Cu^2+^ to Cu^1+^, helps in the formation of oxygen vacancies [69]. This finding was confirmed by the XPS spectra of Cu-2*p* (Fig. 7a), where the strong Cu-2*p* peaks at approx. 932.1 and 952.3 eV for the Cu^1+^ 2*p*_3/2_ and 2*p*_1/2_ peaks and at 933.4 and 954.4 eV for the Cu^2+^ 2*p*_3/2_ and 2*p*_1/2_ peaks in the CCTO–NCAL heterostructure are similar to previously reported values [69, 70]. This reveals that the chemical valences of Cu at the surface of the as-prepared materials are a mixture of valence states + 1 and + 2. The formation of the CCTO–NCAL heterostructure led to the reduction of Cu^2+^ to Cu^1+^, which resulted in the formation of a substantial amount of oxygen vacancies. Subsequently, the deconvolution of the Ti-2*p* peaks into Ti-2*p*_1/2_ and Ti-2*p*_3/2_ is demonstrated in Fig. S8. In pure CCTO, mixed oxidation states of Ti^4+^ and Ti^3+^ are present, with Ti^4+^-2*p*_1/2_ and Ti^4+^-2*p*_3/2_ being the main forms, where Ti^4+^ is dominant in pure CCTO. Furthermore, the heterostructure of CCTO–NCAL formation illustrated an improvement and augmentation in comparison to the pure CCTO. The heterostructure promoted the transition of Ti from Ti^4+^ to Ti^3+^, while an increase in Ti^3+^ promoted the creation of vacancies in CCTO–NCAL [71]. The peaks of Ti^3+^-2*p* are also more pronounced and broader in CCTO–NCAL than pure CCTO, as shown in Fig. S8. Moreover, the density of states of Cu, Ti, and Co has a significant impact on the change in the electronic structure, where the *d* and *s* electrons of Cu and Co play a vital role in the change of Fermi level position. In addition, the electrical conduction of charge carriers in CCTO is ionic (such as oxygen ions via vacancies) or electronic (such as electrons) at certain high temperatures, e.g., at fuel cell operating temperatures. The conductivities of oxides are typically found to be highly dependent upon both carrier concentration and mobility. When the temperature is > 773 K, there is a possibility of a conduction process where oxygen ions participate and the oxygen vacancies are disordered, leading to a lower activation energy for oxygen ions to migrate [59]. Moreover, the DFT calculation also revealed that Ti and Cu played significant role in the density of state near Fermi level in CCTO, NCAL, and CCTO–NCAL, as depicted in Figs. S9a–d and 8a, b. As a result, CCTO delivered considerable peak power densities of 263 and 95 mW cm^−2^ at 550 and 450 °C, respectively. However, a limited number of oxygen ions and limited mobility in the structure and intrinsic resistance of the CCTO material decreased the overall power output and OCV compared to those of the CCTO–NCAL-based device. Indeed, NCAL provides additional ionic conduction as well as increased interfaces for ionic transport. The interaction of the constituent phases produces more oxygen vacancies at the interfaces between CCTO and NCAL due to charge transfer and diffusion, as seen via the oxygen 1*s* spectra (O-1*s*) (Fig. 7b). The available O_*α*_ peak is attributed to the surface oxygen species adsorbed on the oxygen vacancies or to surface defects. The O_β_ peak is assigned to lattice oxygen [72]. The formation of a surface with a high content of active oxygen species resulted in the formation of a substantial amount of oxygen vacancies. Compared with that of separate CCTO, the relative ratio of O_*α*_ and O_*β*_ increased due to the formation of the CCTO–NCAL heterostructure. This increase reveals an increase in the amount of chemisorbed oxygen species in CCTO–NCAL. Hence, all these parameters resulted in the enhancement of the ionic conduction in the CCTO–NCAL electrolyte membrane due to the liberation of the chemisorbed oxygen species of the cell based on the CCTO–NCAL electrolyte at the operating temperatures to expose the surface oxygen vacancies (for oxygen ion transport).

In fact, controlling electronic conduction in fuel cell devices is a prerequisite for fuel cell technology. In this regard, there is a primary need to construct *p*-*n* heterojunctions of CCTO–NCAL to possibly suppress electronic conduction [17, 44]. The constructed interface between CCTO and NCAL increased the dielectric constant by one-fold compared with that of CCTO. It is worth noting that the orientation of the local electric field can help in dipole polarization and charge redistribution, which can directly generate a built-in field at interfaces; consequently, it blocks the electronic flow and increases ionic transport [52, 73, 74]. CCTO–NCAL can have a strong built-in field at the interface due to dipole polarization, which suppresses the electronic flow and enhances ionic transport, while the interface has the intrinsic property of providing a fast path for ionic conduction. The suppression of electronic conduction and enhancement of the fast transport of ionic conduction are also illustrated by rectification verification in the CCTO–NCAL heterojunction by the Schottky junction (SJ), as shown in Fig. 7c, d, which was investigated in air and fuel cell environments. The operation of the fabricated asymmetric fuel cell device of Ni-NCAL/CCTO–NCAL in air followed Ohm’s law, which provided evidence that there was no junction between Ni-NCAL and the semiconductor heterostructure, as suggested by Ohm’s law, as shown in Fig. 7c. Furthermore, the same asymmetric fuel cell device was operated under fuel cell conditions, where the device was allowed to decrease for 30 min. As the resistance decreases and the current increases, the diode behavior increases, and with time, the Schottky junction between the Ni metal formed on the surface of Ni-NCAL after reduction and the CCTO–NCAL semiconductor heterostructure is formed (see Fig. 7d). As mentioned above, the formation of a junction helps in two different aspects, i.e., the SJ blocks the electronic conduction as well as the formation of a built-in electric field that supports the fast transport of ionic conduction. Therefore, the formation of a junction is also the main aspect for enhancing the power output and maintaining the OCV of the device without degradation in the CCTO–NCAL electrolyte-based fuel cell device.

### First Principle Analysis of Heterostructure Formation and Its Electronic Properties

In general, dielectric materials possess the intrinsic characteristics of a wide bandgap, and this study provides evidence for tuning their optical and electrical properties. Therefore, considering the intrinsic properties of CCTO dielectric materials, this study provides a detailed investigation of tuning their energy band structure according to desired applications. Herein, we designed a CCTO–NCAL heterostructure by manipulating energy bands to understand and demonstrate the mechanism of energy band engineering. To complement the measurements and to gain microscopic insight into the electronic properties of individual NCAL and CCTO (related to heterostructure formation), the orbital projected density of states (PDOS) and the orbital projected band structure were calculated and discussed. A similar part of these calculations was reproduced for the heterostructure as a whole. Heterostructure formation refers to the engineering of the interface, and the sole purpose of energy band alignment at interfaces is the formation of oxygen vacancies by the interaction of particles belonging to two different structured semiconductor oxide materials with different conducting properties. In this regard, the total and partial density of states (TDOS and PDOS) and the energy band structure for the CCTO, NCAL, and CCTO–NCAL heterostructures at the (400) and (110) planes are presented in Figs. S9 and 8. Each PDOS of CCTO, NCAL, and CCTO–NCAL predicts spin-up and spin-down orbitals with sharp peaks for each element (i.e., Ca-4*s*, O-2*p*, Cu-3*d*, Ti-3*d*, Ni-3*p*, Co-3*d*, Al-3*p*, and Li-2*s*), where the bands are near the Fermi level. Since both compounds exhibit spin asymmetry (splitting) and CCTO is known to form more than one spin-ordered phase [75], the geometry had to be refined for each spin configuration. For each spin configuration, the resulting band structure and DOS are projected into their orbital components for each spin orientation. In addition to VASP, the SUMO library was used to generate K-space paths and supplementary roles [76]. Since CCTO crystals are known to form more than one spin-ordered phase, the electronic and spin degrees of freedom must be treated separately in the following, in our case by a two-component method. The CCTO crystal contains two phases, one spin symmetric and one asymmetric phase. The NCAL converged on the spin asymmetric phase. These can be seen in the orbital projection plots below, which were generated using the SUMO library after supplying densities and wave functions obtained from VASP. For clarity, only the three most prominent orbitals are displayed. Their sum represents almost all electronic states in this energy range (close to the Fermi level). To explore the electronic properties of a crystal, it is necessary to know the energy levels of the occupied and virtual orbitals for k-points in reciprocal space. To cover this space, one chooses a path through the phases of the reciprocal lattice that covers all the important corners. Owing to the shape of the cell, it is not possible to draw a single trajectory; therefore, it must be split in two for all the structures considered. For the spin-symmetric anti-ferromagnetic ordered CCTO, there is no point in displaying the spin-projected band structure since it is twofold degenerate. Instead, the orbital projected band structure is shown in Fig. S9a, b, where the color of the bands is a mixture of the three fundamental colors representing the three most prominent orbitals.

For the spin asymmetric cases, i.e., NCAL and the metastable phase of CCTO, the behavior around the Fermi level was analyzed using the spin-projected band structure. Interestingly, for the spin asymmetric case of CCTO (depicted in Fig. S9c, d), conduction near the Fermi point is almost exclusively driven by a single spin orientation. This phenomenon is even more pronounced for NCAL, which behaves exactly like the spin half metal presented in Fig. S9e, f.

### Theoretical Analysis of Heterostructure Formation

**Fig. 8:**
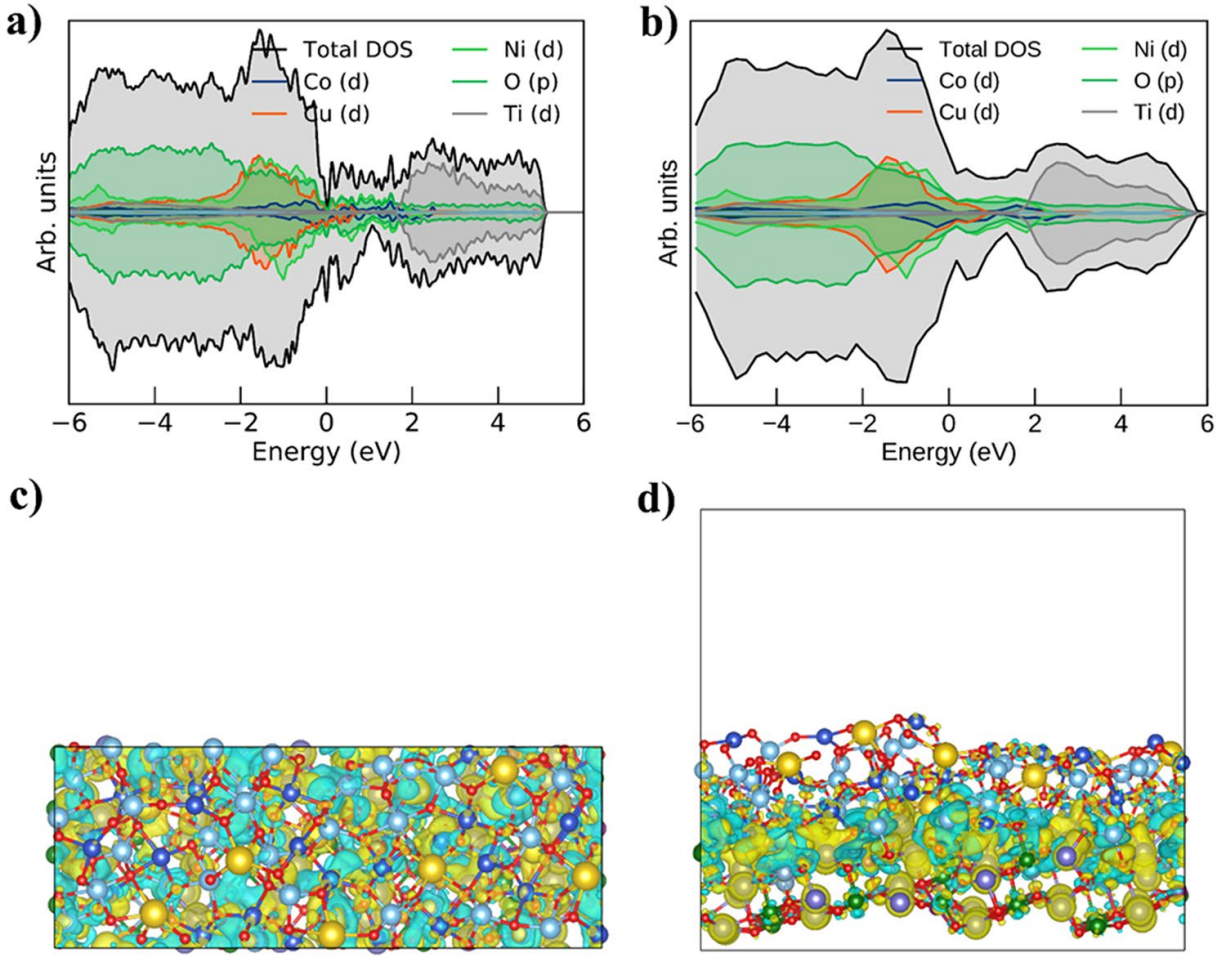
**a**, **b** Density of states of CCTO(422)/NCALO(101) heterostructure composite of specifically Cu-*d*, O-*p*, Ti-d, Ni-*d*, and Co-*d*. Charge density difference of CCTO(422)/NCALO(101) heterostructure. **c**, **d** Top and side view of the relaxed and calculated charge accumulation and depletion indicated by yellow and cyan area, respectively. Isosurface level was set at 0.002 e Å^−3^

The primary objective of this study was to construct a CCTO–NCAL heterostructure. To this end, the top and side views of the CCTO and NCAL layers at the (422) and (101) planes were considered. This led to the formation of an ideal interface that forces toward the Fermi level, as depicted in Fig. 8. Following the successful construction of the heterostructure, the total and partial density of states (TDOS and PDOS) at the (422) and (101) planes were calculated and are presented in Fig. 8a, b. The spin-up and spin-down orbitals of the partial density of states (PDOS) of the CCTO–NCAL heterostructure exhibit peaks corresponding to each constituent element (Ca, Cu, Ti, O, Ni, Co, Al, and Li). The energy bands are shifted toward the Fermi level, as depicted in Fig. 8a, b, in comparison to those of individual CCTO and NCAL. The TDOS of the CCTO–NCAL heterostructure is substantially closer to the Fermi level (than that of the individual components), providing evidence that the designed heterostructure, as confirmed by the experimental results. The valence band of CCTO is primarily composed of Cu-3*d* atomic orbitals, while the conduction band is formed by the 3*d* atomic orbitals of Ti. Nevertheless, the presence of Ti affects the overall electronic properties of the entire CCTO sublattice, resulting in the formation of defects [77]. In contrast, Ni-3*p* and Co-3*d* are solely responsible for the construction of the band in NCAL.

Furthermore, the charge density difference in the CCTO–NCAL heterostructure was calculated via the iso-surface level of the charge density difference, where the isosurface level in the CCTO–NCAL heterostructure was fixed at 0.01 e Å^−3^ (Fig. 8c, d). The electronegativity of CCTO–NCAL follows this order (based on the Pauling exclusion principle); in addition, the Bader charge analysis showed a charge transformation of 3.49 |e| (from NCAL to CCTO).

### Unrevealing Energy Band Bending Characteristics of Electrolyte for Fuel Cell

**Fig. 9:**
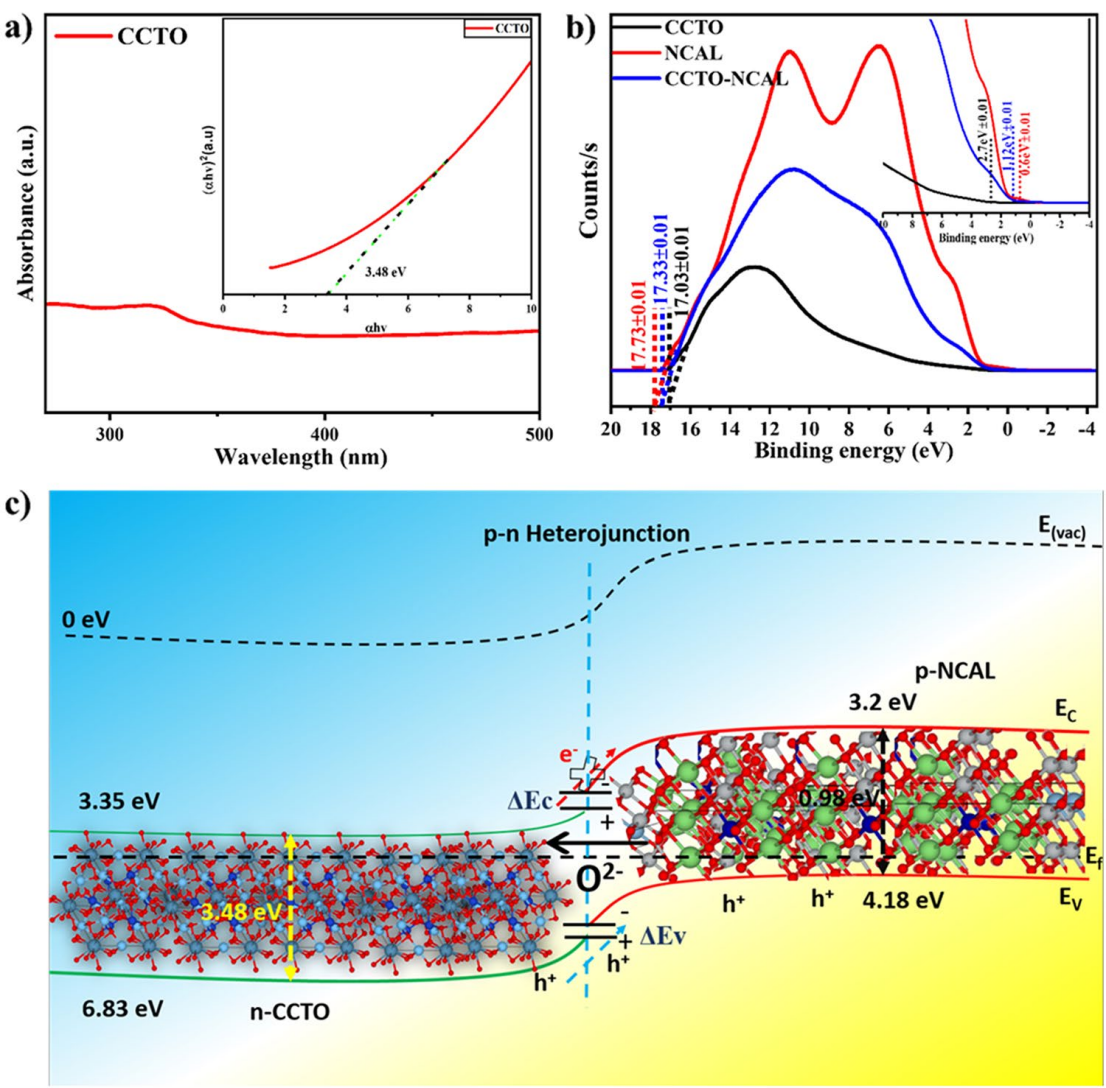
**a** UV–Vis spectra of CCTO, complete spectrum and energy optical gap. **b** UPS spectra cutoff energy region, and onset energy region of CCTO, NCAL, and CCTO–NCAL. **c** Energy band diagram of fuel cell CCTO–NCAL heterostructure obtained according to UPS results and energy band gap

The exploration of energy band engineering at the interface of heterostructure materials, including dielectric materials modified via the introduction of semiconductor oxide materials, represents an intriguing mechanism. In this context, UV–Vis spectroscopy and UPS were employed to obtain the respective valence band maxima and energy bandgaps, with the objective of determining and demonstrating the energy band alignment. The resulting values are presented in Fig. 9. It is first necessary to understand the principles of energy band theory, which is essential for the successful alignment of energy bands at the heterostructure interface of two different types of materials at the nanoscale [68, 78]. Following the principles of energy band theory, which are applicable to the design of fuel cells and the construction of energy band structures, a typical *n*-type semiconductor with a C_B_ greater than −4.5 eV RVE was used as the electrolyte. Subsequently, a heterostructure of *n*-type CCTO and *p*-type NCAL is employed. A heterostructure comprising a wideband bandgap of 3.48 eV (CCTO) and a narrow bandgap (NCAL) of 0.98 eV was employed in this study [79]. From the results of ultraviolet photoelectron spectroscopy (UPS) and UV‒Vis spectroscopy (UV‒Vis), we calculated the energy band gaps and the valence band maxima (depicted in Fig. 9a, b), which were then utilized for the energy band diagram of a CCTO–NCAL heterostructure electrolyte fuel cell (Fig. 9c). According to Eq. (5):

5 $$\varphi = 21.2 {\mathrm{eV}} - \left( {E_{{{\mathrm{cutoff}}}} - E_{{{\mathrm{onset}}}} } \right)$$

where *E*_cutoff_ is the cutoff energy and Eonset is the onset energy from UPS (Fig. 9b), the valence band (*V*_B_) for CCTO and NCAL were calculated to be 6.83 and 4.18 eV, respectively. Furthermore, the *C*_B_ values for the two materials were calculated from the optical energy gap (*E*_g_) obtained from the absorbance spectra of each constituent, as presented in Fig. 9a, b. The cutoff energy at higher binding energy of CCTO and NCAL were presented in supporting information (Fig. S10a, b). The built energy band diagram follows the energy band theory and even introduces a dielectric CCTO that behaves like an *n*-type semiconductor with a wide band gap, thereby creating a successful heterostructure with NCAL [80]. Consequently, it can be reasonably assumed that a considerable power output can be guaranteed from the designed heterostructure, as evidenced by the fuel cell performance data presented above.

## Conclusions

For the first time, a dielectric CCTO material was utilized as an ion-conducting electrolyte for LT-SOFCs. This is an inaugural report on the introduction of a dielectric material as an electrolyte membrane in LT-SOFCs. The electrical properties of CCTO exhibited notable differences in the presence of air and fuel cell atmospheres. For instance, the electrical conductivity of CCTO in air at 550 °C was 2.2 × 10^–3^ S cm^−1^, while the ionic conductivity under fuel cell conditions reached 0.098 S cm^−1^. CCTO was subsequently coupled with NCAL to form a CCTO–NCAL heterostructure, where the conductivity was increased to 1.01 × 10^–2^ S cm^−1^ in air and the ionic conductivity was increased to 0.15 S cm^−1^ under fuel cell conditions. The morphology, structure, composition, photoelectrochemical properties, and operation of the heterostructure were comprehensively characterized both theoretically and experimentally under fuel cell conditions. Based on the enormous apparent dielectric constant and extra EIS polarization resistance, a new interfacial polarization was derived for the charge transfer of electrons between grain boundaries and at the particle/grain interface, which hinders electronic conduction in the heterostructure. CCTO–NCAL showed a significantly greater dielectric constant than CCTO. This phenomenon is ascribed to the introduction of NCAL to CCTO, which leads to higher values of *ε*_*r*_, thus reducing electron leakage in terms of high OCVs. This resulted in the tuning of ionic conduction, and considerable oxide ion conduction was achieved in the CCTO–NCAL electrolyte. The heterostructure interface is responsible for the dielectric property change while simultaneously providing a high conduction path for oxide ions. This work opens a new pathway for preparing dielectric materials (e.g., CCTO) and their heterostructures (e.g., NCAL) that are promising for use as electrolytes in LT-SOFCs.

## Supplementary Information

Below is the link to the electronic supplementary material.

Supplementary file1 (DOCX 5039 KB)
