# Correction: Alternative Strategy for Development of Dielectric Calcium Copper Titanate-Based Electrolytes for Low-Temperature Solid Oxide Fuel Cells

**DOI:** 10.1007/s40820-026-02081-3

**Published:** 2026-02-04

**Authors:** Sajid Rauf, Muhammad Bilal Hanif, Zuhra Tayyab, Matej Veis, M. A. K. Yousaf Shah, Naveed Mushtaq, Dmitry Medvedev, Yibin Tian, Chen Xia, Martin Motola, Bin Zhu

**Affiliations:** 1 College of Mechatronics and Control Engineering, Shenzhen University, Shenzhen, 518060 People’s Republic of China; 2 Department of Inorganic Chemistry, Faculty of Natural Sciences, Comenius University Bratislava, Ilkovicova, 684215 Bratislava, Slovakia; 3 Energy Storage Joint Research Center, School of Energy and Environment, Southeast University, Nanjing, 210096 People’s Republic of China; 4 Hydrogen Energy Laboratory, Ural Federal University, 620002 Ekaterinburg, Russia; 5 Laboratory of Electrochemical Devices Based on Solid Oxide Proton Electrolytes, Institute of High Temperature Electrochemistry, 620066 Ekaterinburg, Russia; 6 School of Microelectronics, Hubei University, Wuhan, 430062 People’s Republic of China

**Correction to: Nano-Micro Letters (2025) 17:13** 10.1007/s40820-024-01523-0

Following publication of the original article [1], some errors were identified by the authors, which were not observed during proof reading.

- The authors have added additional scale values even in the presence of measured (real) scale bar and line resolution on SEM images to make them obvious on the real (raw) images presented in Fig. 3(g–i) and similarly, in Fig. S7. However, they would like to have SEM images only with measured (real) scale bar and line resolution on real (raw) images in Fig. 3(g–i) and Fig. S7 in order not to mislead the scientific society and the young researchers in this field.
- The scale of EIS on x-axis and y-axis in Fig. 5(c–d) and Fig. 6(a–b) has been mistakenly written as Ω cm^−2^, while the correct unit is Ω cm^2^. Unit and scale are corrected. The authors would like to remove the indication of fitted curve from Fig. 5(c–d); similarly, Fig. 6(a–b) had no fitted curve indication in published version.
- There is a typo error in the value of Q1 at 550 °C in Table 3, where the correct value is 5.97E-4 instead of 5.97E-14. The correct value of n is 6.73E-01 instead of 6.73E-02 at 500 °C in Table 3.
- The authors have identified a wrong funding number (51736006) in the funding source in the acknowledgments section; they want to remove it.

The updated Figs. 3, 5, 6 and Table 3 have been provided in this correction. The Acknowledgements and Supplementary file have been updated.

The incorrect Fig. 3 is:

**Fig. 3:**
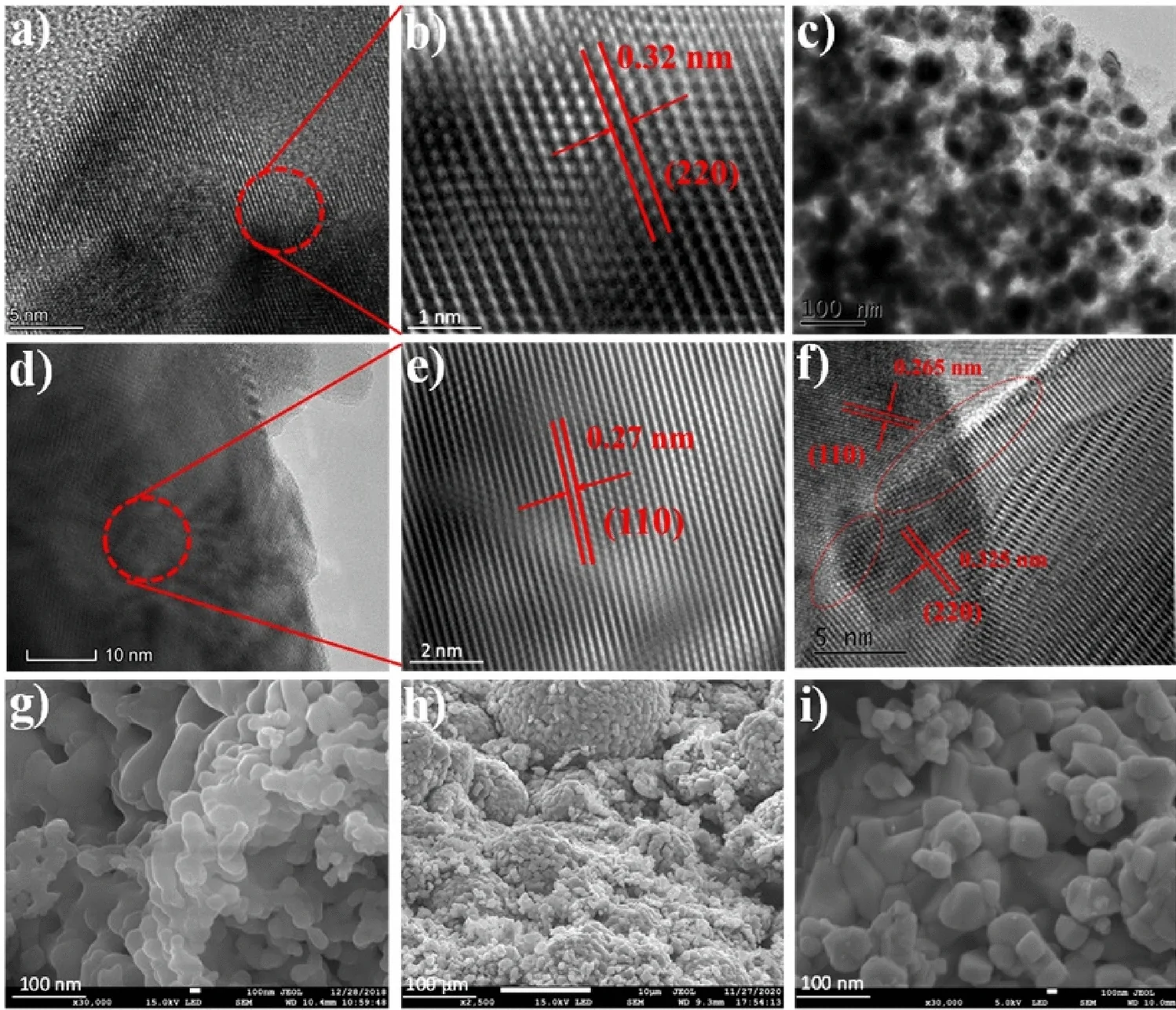
**a**–**f** High-resolution transmission electron microscope images representing the microstructure included the calculated lattice spacing and particle distribution with hetero-interfaces at the particles-level of CCTO, NCAL, and CCTO–NCAL heterostructure. **g**–**i** Field emission-scanning electron microscopy images depicting: the surface morphology and particles distribution of the CCTO, NCAL, and CCTO–NCAL heterostructure

The correct Fig. 3 is:

**Fig. 3:**
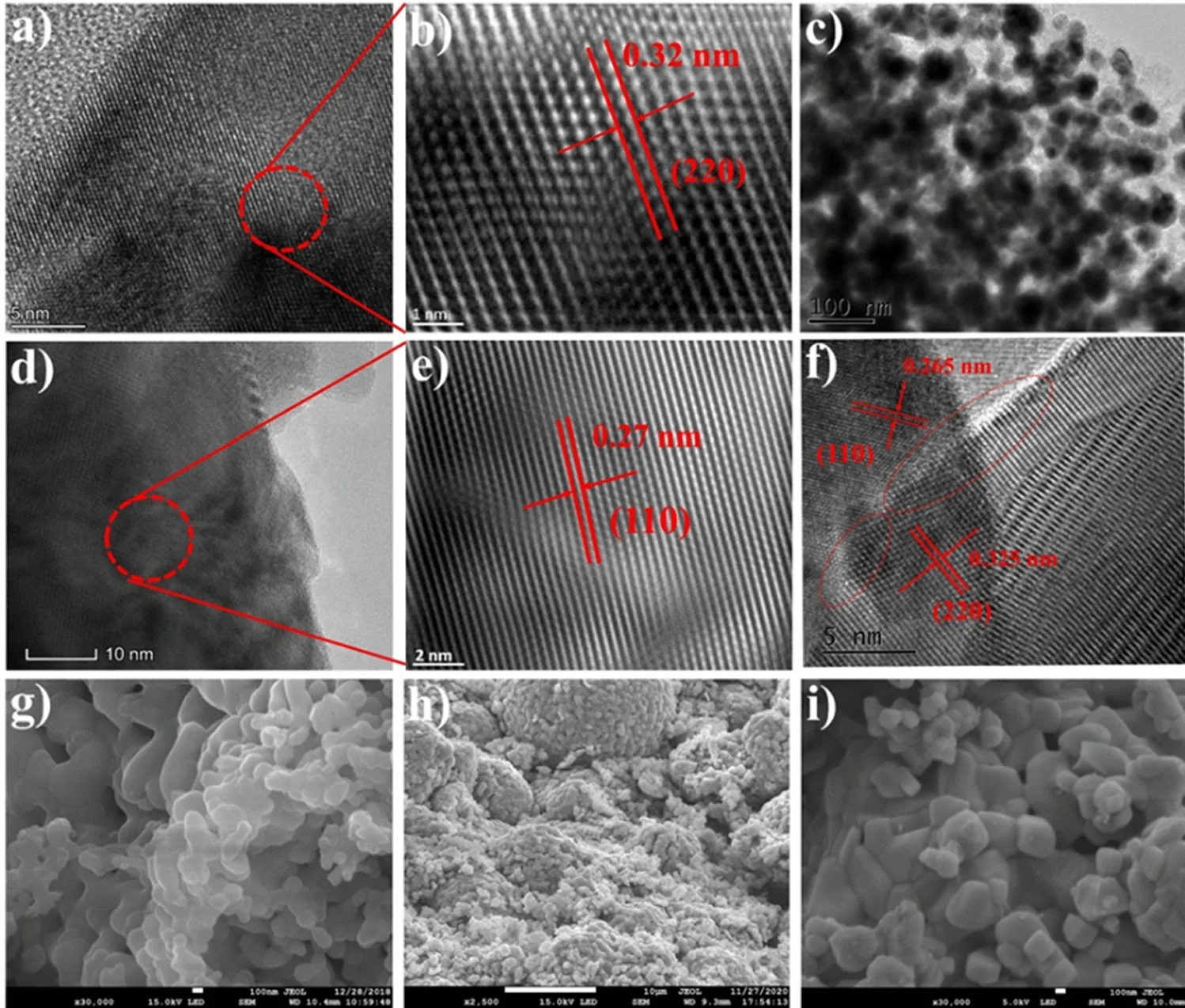
** a–f** High-resolution transmission electron microscope images representing the microstructure included the calculated lattice spacing and particle distribution with hetero-interfaces at the particles-level of CCTO, NCAL, and CCTO–NCAL heterostructure. **g–i** Field emission-scanning electron microscopy images depicting: the surface morphology and particles distribution of the CCTO, NCAL, and CCTO–NCAL heterostructure.

The incorrect Fig. 5 is:

**Fig. 5:**
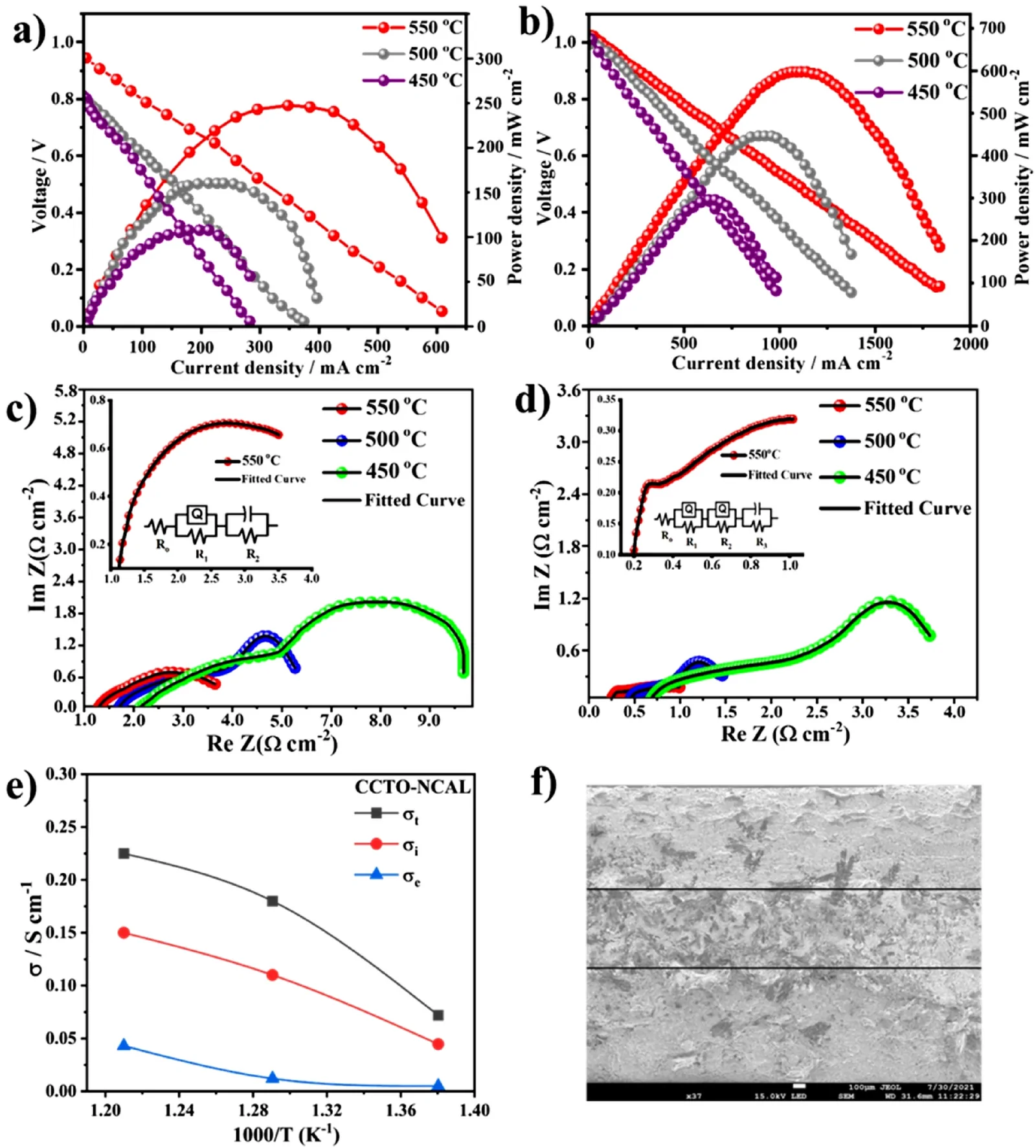
Fuel cell performance in terms of current density and voltage (*I-V*) and current density and power density (*I–P*) of the **a** CCTO and **b** CCTO–NCAL heterostructure electrolyte cells in the H_2_/air environments at operational temperature of 550–450 °C. Impedance spectra of **c** CCTO membrane cell and **d** CCTO–NCAL electrolyte membrane cell at different temperatures and their fitted circuits under H_2_/Air environment. **e** Total, ionic, and electronic conductivity contribution in CCTO–NCAL electrolyte membranes in fuel cell conditions at different temperatures. **f** SEM image of cross-sectional view of the fuel cell device with architecture of Ni-NCAL/CCTO–NCAL/NCAL-Ni cell

The correct Fig. 5 is:

**Fig. 5:**
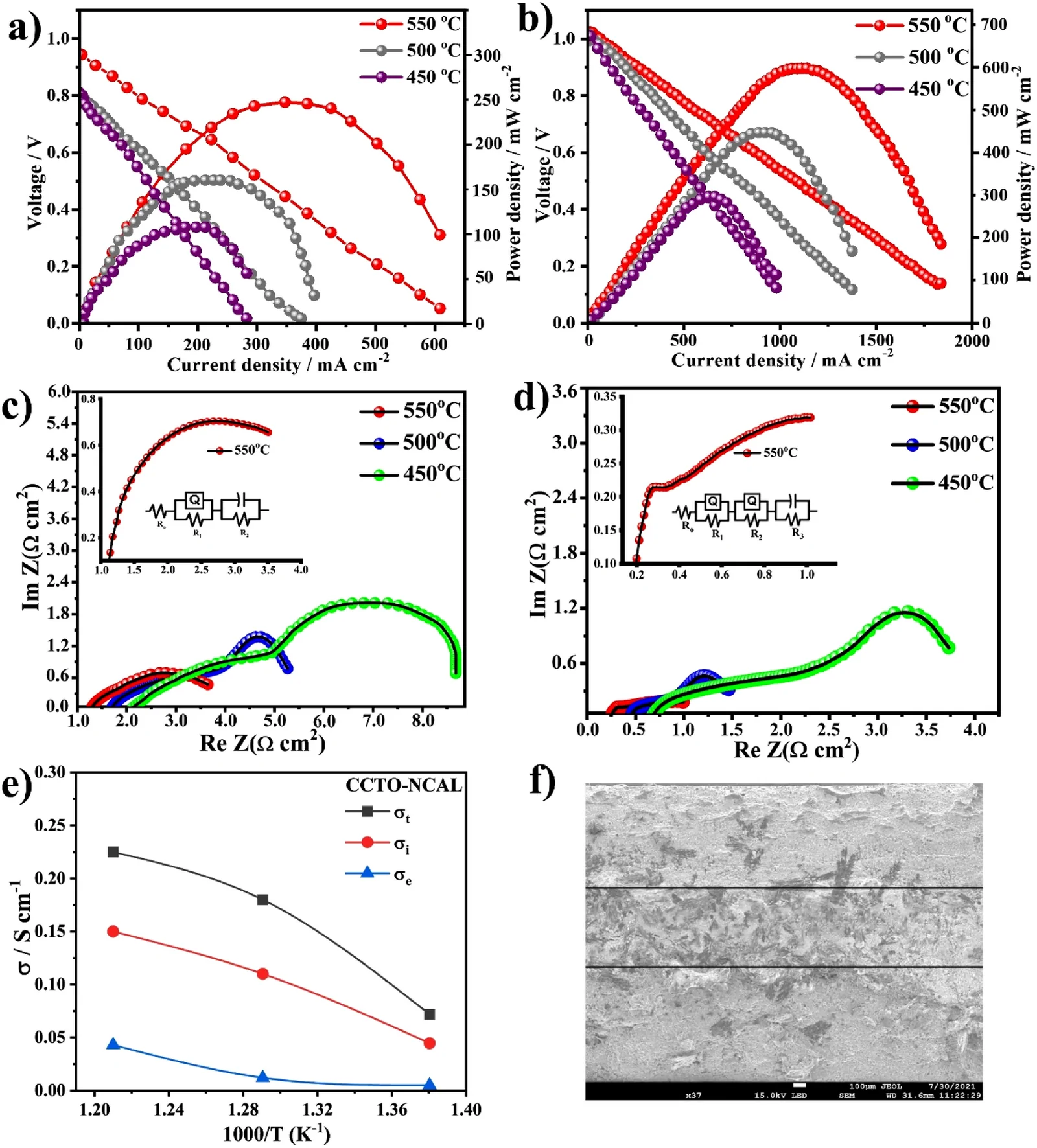
Fuel cell performance in terms of current density and voltage (*I-V*) and current density and power density (*I–P*) of the **a** CCTO and **b** CCTO–NCAL heterostructure electrolyte cells in the H_2_/air environments at operational temperature of 550–450 °C. Impedance spectra of **c** CCTO membrane cell and **d** CCTO–NCAL electrolyte membrane cell at different temperatures and their fitted circuits under H_2_/Air environment. **e** Total, ionic and electronic conductivity contribution in CCTO–NCAL electrolyte membranes in fuel cell conditions at different temperatures. **f** SEM image of cross-sectional view of the fuel cell device with architecture of Ni-NCAL/CCTO–NCAL/NCAL-Ni cell

The incorrect Fig. 6 is:

**Fig. 6:**
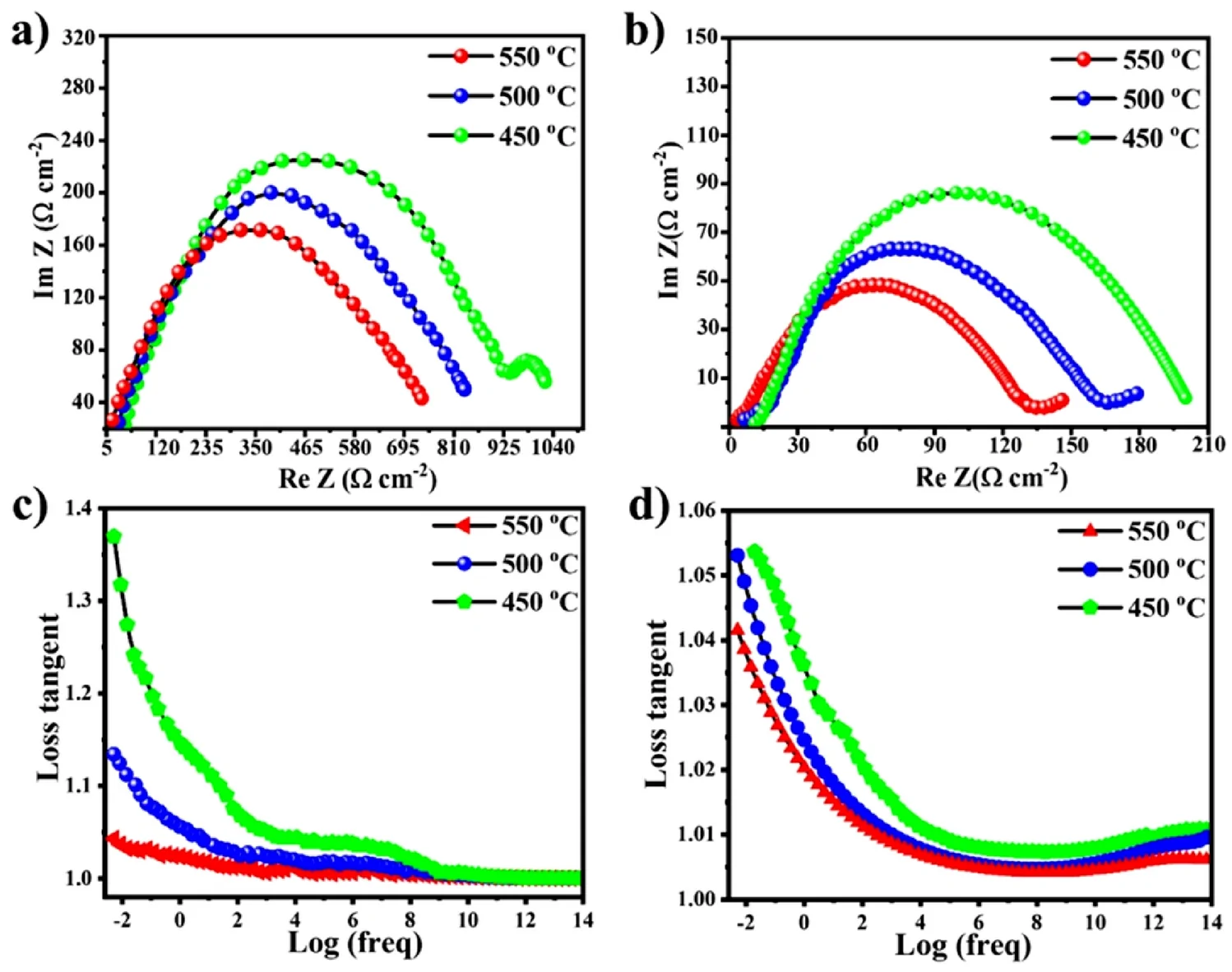
Impedance spectra of **a** CCTO and **b** CCTO–NCAL using Ag electrodes; frequency dependence of loss tangent (tan *δ*) for **c** CCTO and **d** CCTO–NCAL membrane at selected temperatures

The correct Fig. 6 is:

**Fig. 6:**
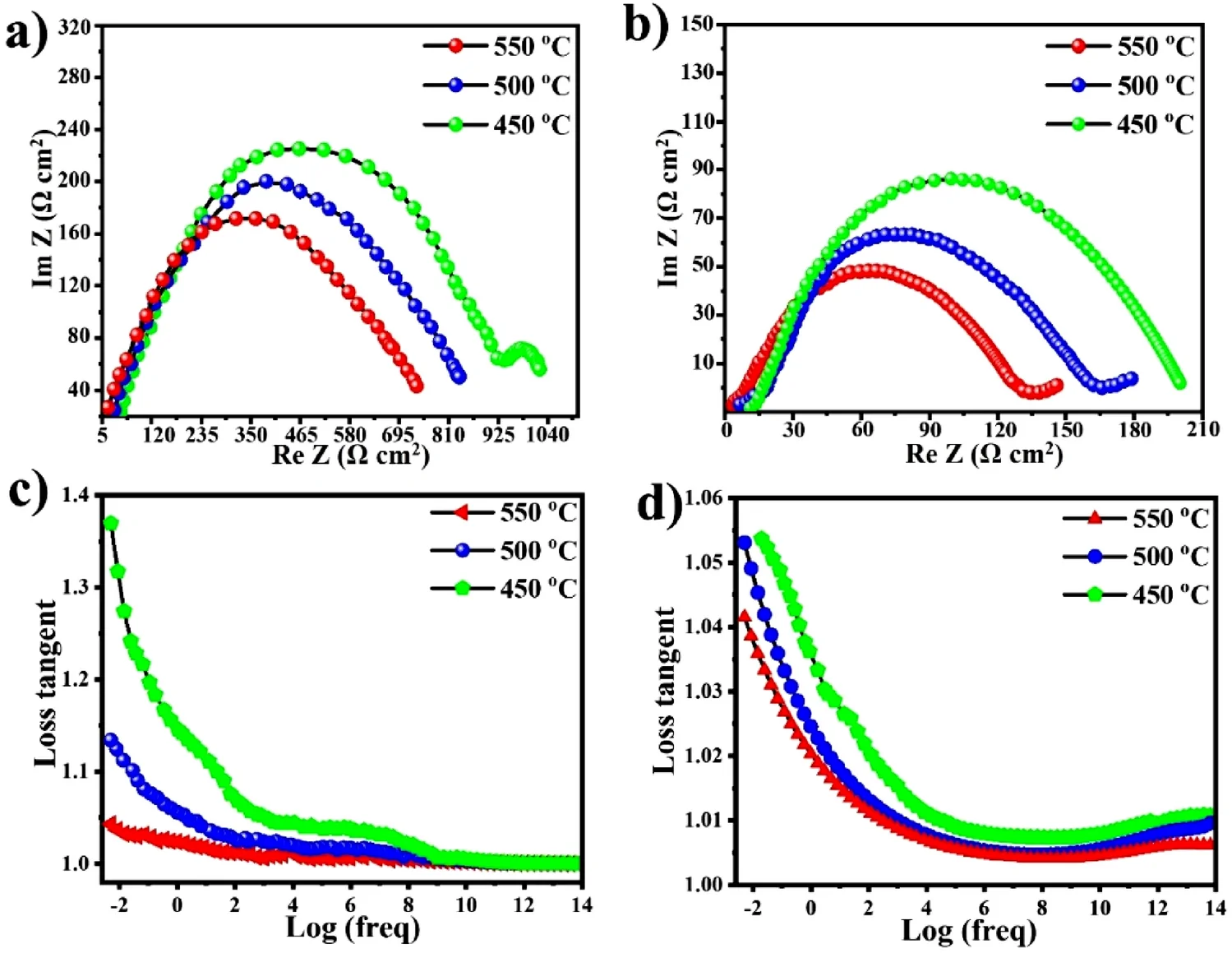
Impedance spectra of a CCTO and b CCTO–NCAL using Ag electrodes; frequency dependence of loss tangent (tan δ) for c CCTO and d CCTO–NCAL membrane at selected temperatures

The incorrect Table 3 is:

**Table 3:** EIS fitted data obtain for CCTO and CCTO-NCAL using NCAL an electrodes electrolyte membranes using ZSimpWin software, where n is frequency power, n [0 < n < 1]

Composition (°C)	*R*_1_ (Ω cm^2^)	*R*_2_ (Ω cm^2^)	*Q*_1_ (CPE_1_) *Y*_o_[(*S*–*s*)^*n*^ cm^−2^]	Q_2_ (CPE_2_) *Y*_o_[(*S*–*s*)^*n*^ cm^−2^]	*R*_3_ (Ω cm^2^)	*R*_4_ (Ω cm^2^)	*C*_1_ (F cm^–2^)	*n*
CCTO								
550 °C	1.201	1.412	5.28E-03		0.921		2.83E-01	3.36E-01
500 °C	1.704	2.312	1.63E-02		1.411		1.13E-01	1.66E-01
450 °C	2.101	2.842	6.90E-03		4.845		5.25E-07	2.10E-01
CCTO-NCAL								
550 °C	0.201	0.212	5.97E-14	0.1251	0.66	–	1.12E-05	1.12E-01
500 °C	0.414	0.505	2.887	0.2355	0.08	0.4	1.04E-05	6.73E-02
450 °C	0.612	0.388	0.05023	0.009546	1.402	1.25	0.1647	1.26E-01

The correct Table 3 is:

**Table 3:** EIS fitted data obtain for CCTO and CCTO-NCAL using NCAL an electrodes electrolyte membranes using ZSimpWin software, where n is frequency power, n [0 < n < 1]

Composition (^o^C)	R_1_ (Ω cm^2^)	R_2_ (Ω cm^2^)	Q_1_ (CPE_1_) Y_o_[(S–s)^n^ cm^−2^]	Q_2_ (CPE_2_) Y_o_[(S–s)^n^ cm^−2^]	R_3_ (Ω cm^2^)	R_4_ (Ω cm^2^)	C_1_ (F cm^–2^)	n
CCTO								
550	1.201	1.412	5.28E-03		0.921		2.83E-01	3.36E-01
500	1.704	2.312	1.63E-02		1.411		1.13E-01	1.66E-01
450	2.101	2.842	6.90E-03		4.845		5.25E-07	2.10E-01
CCTO-NCAL								
550	0.201	0.212	5.97E-4	0.1251	0.66	–	1.12E-05	1.12E-01
500	0.414	0.505	2.887	0.2355	0.08	0.4	1.04E-05	6.73E-01
450	0.612	0.388	0.05023	0.009546	1.402	1.25	0.1647	1.26E-01

The authors sincerely apologize for such minor oversights. These minor human mistakes that do not affect the results or conclusions of the work, but correcting them will ensure clarity and accuracy for readers.

The original article [1] has been corrected.

## References

[1] S. Rauf, M.B. Hanif, Z. Tayyab et al., Alternative strategy for development of dielectric calcium copper titanate-based electrolytes for low-temperature solid oxide fuel cells. Nano-Micro Lett. **17**, 13 (2025). 10.1007/s40820-024-01523-0 10.1007/s40820-024-01523-0 PMC11427654 39325255

