# Supplementary material for: Alternative Strategy for Development of Dielectric Calcium Copper Titanate-Based Electrolytes for Low-Temperature Solid Oxide Fuel Cells
Source: Nanomicro Lett. 2024 Sep 26;17:13. doi: 10.1007/s40820-024-01523-0 (PMC11427654; doi:10.1007/s40820-024-01523-0)
Supplement: Supplementary file 1 — Supplementary file1 (DOCX 5039 KB) [file 40820_2024_1523_MOESM1_ESM.docx]

Supporting Information for

**Alternative Strategy for Development of Dielectric Calcium Copper Titanate Based Electrolytes for Low Temperature Solid Oxide Fuel cells**

Sajid Rauf^1^, Muhammad Bilal Hanif^2^, Zuhra Tayyab^1^, Matej Veis^2^, MA.K. Yousaf Shah^3^, Naveed Mushtaq^3^, Dmitry Medvedev^4, 5,^*, Yibin Tian^1,^*, Chen Xia^6^, Martin Motola^2^, Bin Zhu^3,^*,

^1^ College of Mechatronics and Control Engineering, Shenzhen University, Shenzhen 518060, P. R. China

^2^ Department of Inorganic Chemistry, Faculty of Natural Sciences, Comenius University Bratislava, Ilkovicova 684215 Bratislava, Slovakia

^3^ Energy Storage Joint Research Center, School of Energy and Environment, Southeast University, Nanjing 210096, P. R. China

^4^ Hydrogen Energy Laboratory, Ural Federal University, 620002, Ekaterinburg, Russia

^5^ Laboratory of Electrochemical Devices Based on Solid Oxide Proton Electrolytes, Institute of high Temperature Electrochemistry, 620066, Ekaterinburg, Russia

^6^ School of Microelectronics, Hubei University, Wuhan 430062, P. R. China

*Corresponding authors. E-mail: [zhu_bin@seu.edu.cn](mailto:zhu_bin@seu.edu.cn) (Bin Zhu); [ybtian@szu.edu.cn](mailto:ybtian@szu.edu.cn) (Yibin Tian); [dmitrymedv@mail.ru](mailto:dmitrymedv@mail.ru) (Dmitry Medvedev)

**Supplementary Figures**


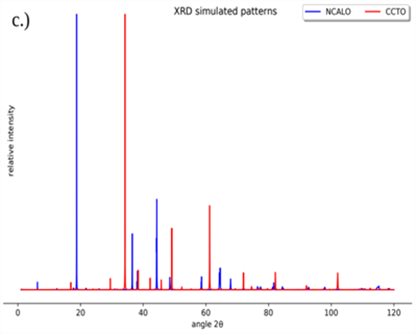


**Fig. S1** Simulated XRD data of NCAL and CCTO


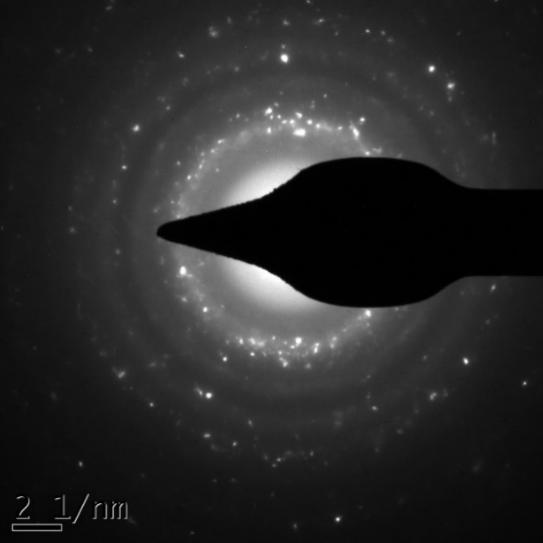


**Fig. S2** The selected area (electron) diffraction (SAED) of the CCTO-NCAL heterostructure


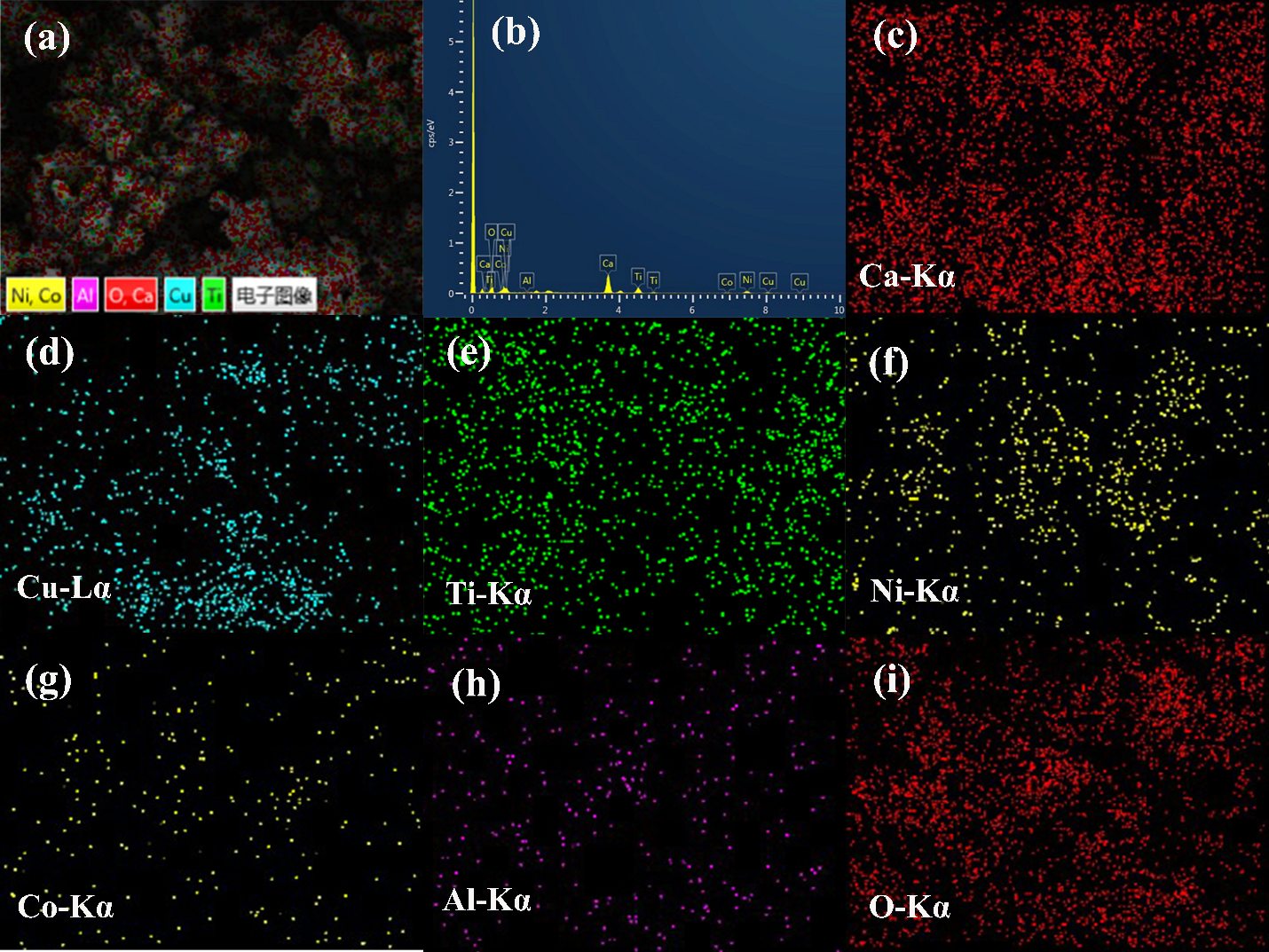


**Fig. S3** SEM assisted EDS mapping, Mix color mapping and elemental mapping of CCTO-NCAL (**a-b**); Each respective elements mapping present in the CCTO-NCAL according to compositional ratio (**c-i**)


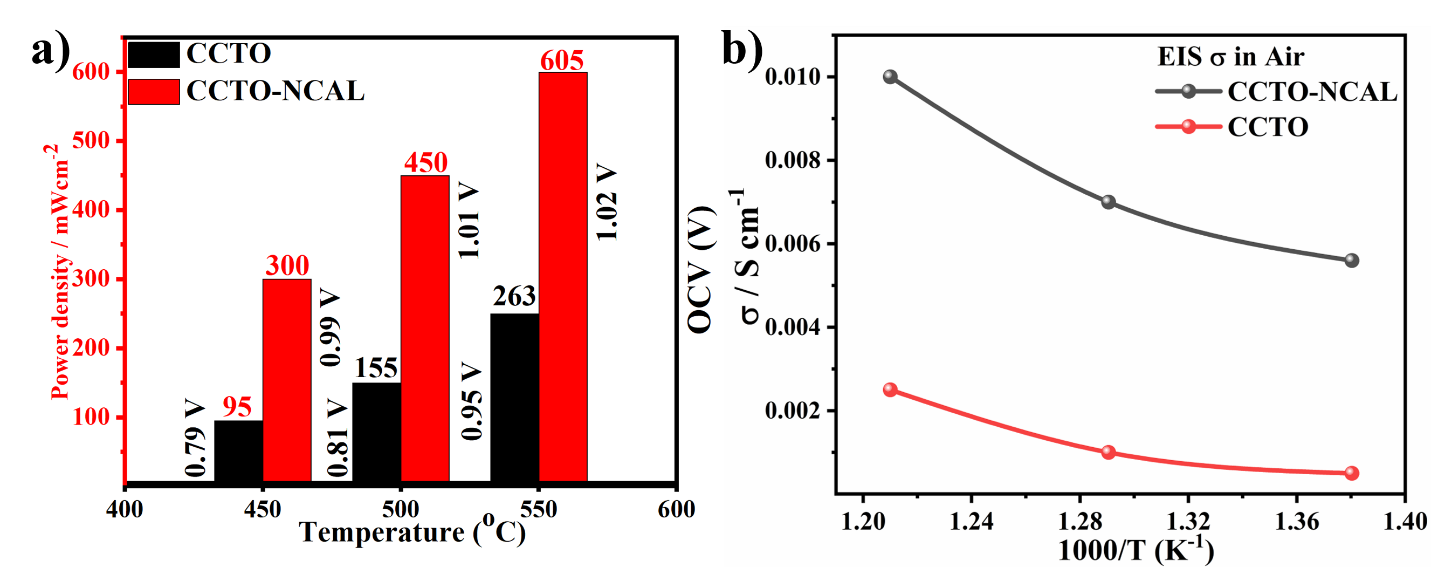


**Fig. S4** Histogram to compare the fuel cell power density and OCV against respective operational temperature


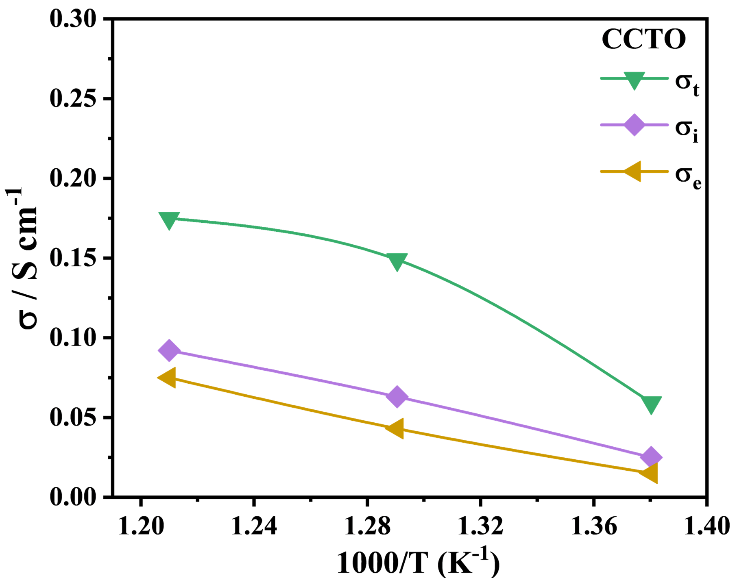


**Fig. S5** Total, ionic and electronic conductivity contribution in CCTO electrolyte membranes in fuel cell conditions at different temperatures


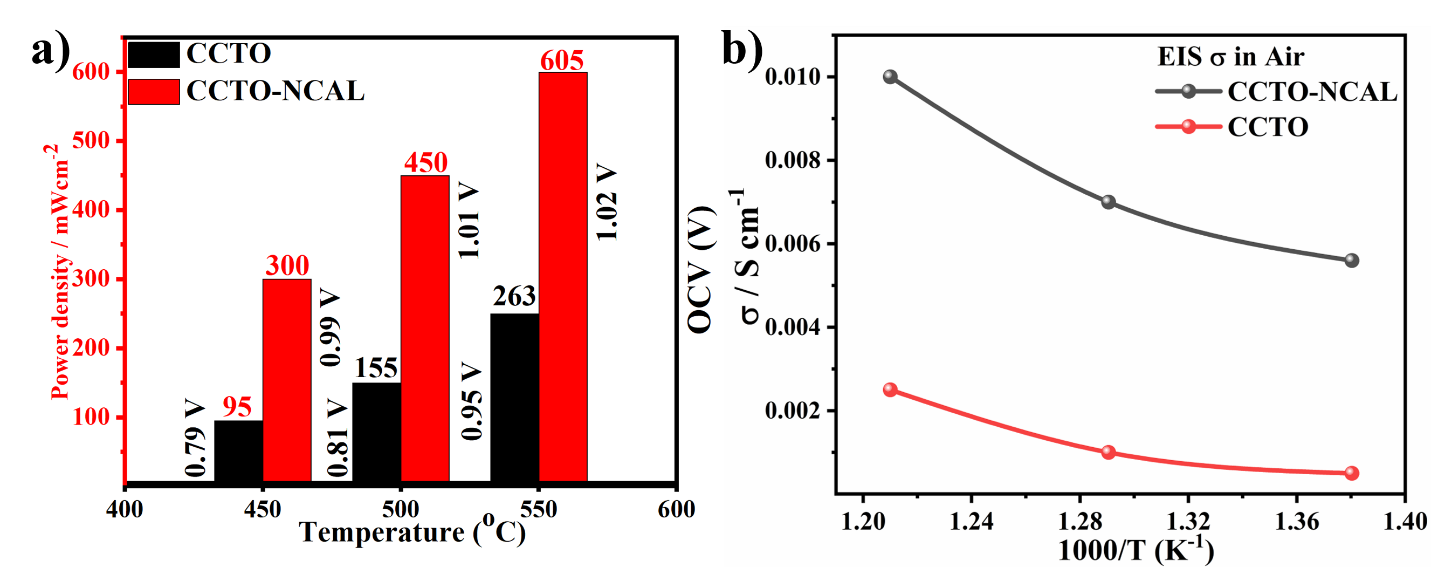


**Fig. S6** The total conductivity obtained from the electrochemical impedance spectroscopy performed under air environment


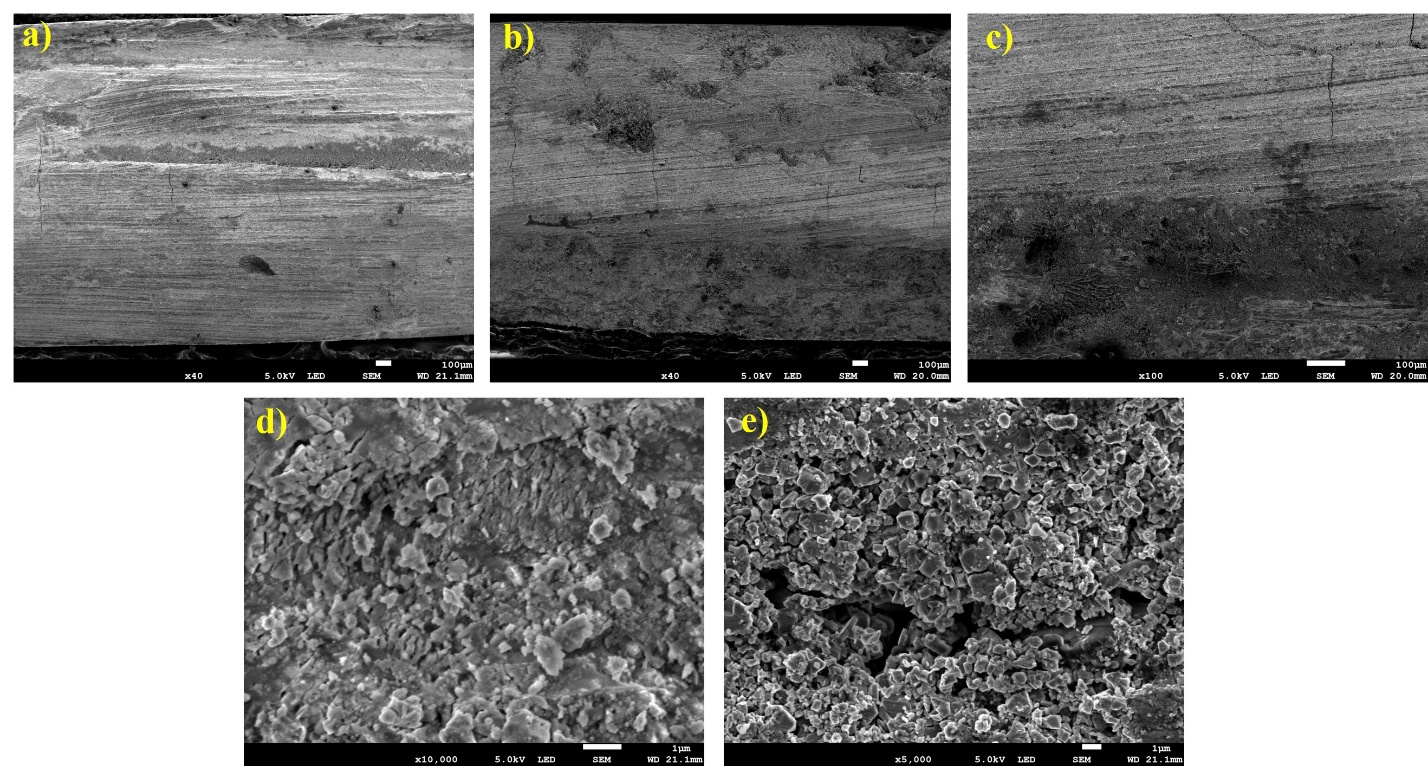


**Fig. S7** SEM images of Cross-sectional and electrolyte of fuel cell before and after testing (**a-b**); the interface formed between electrolyte and anode after testing fuel cell (**c**); morphology of CCTO-NCAL acted as electrolyte (**d**); and NCAL electrode (**e**) in fuel cell after operation


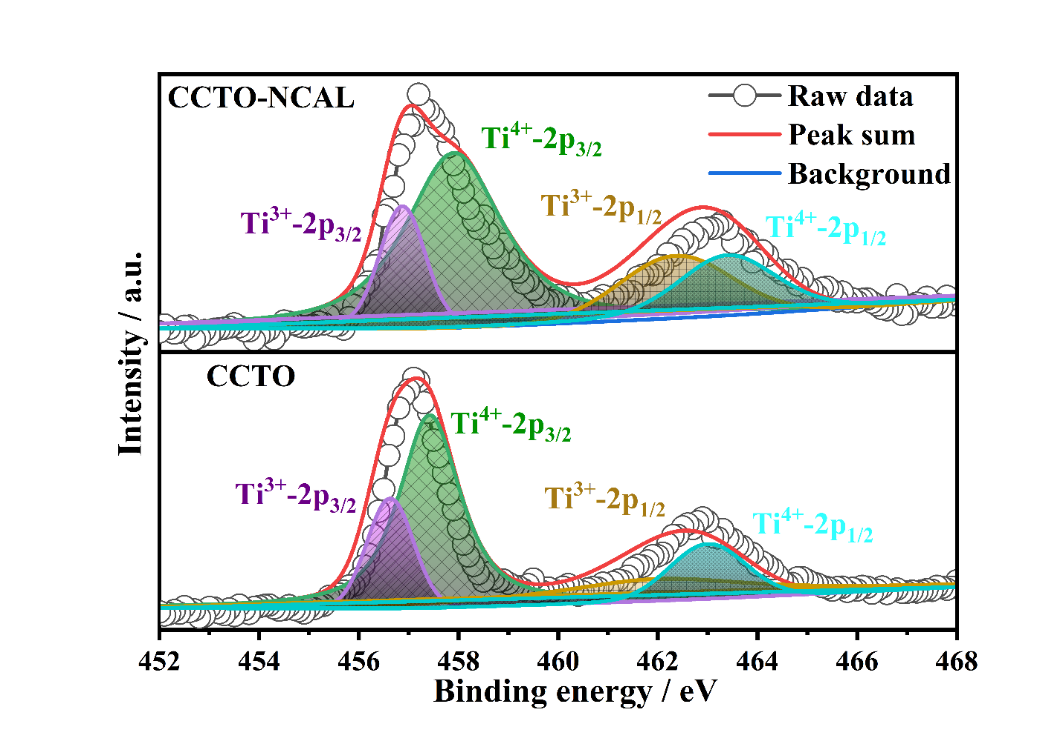


**Fig. S8** XPS spectra of de-convoluted Ti-2p spectra of CCTO and CCTO-NCAL heterostructure


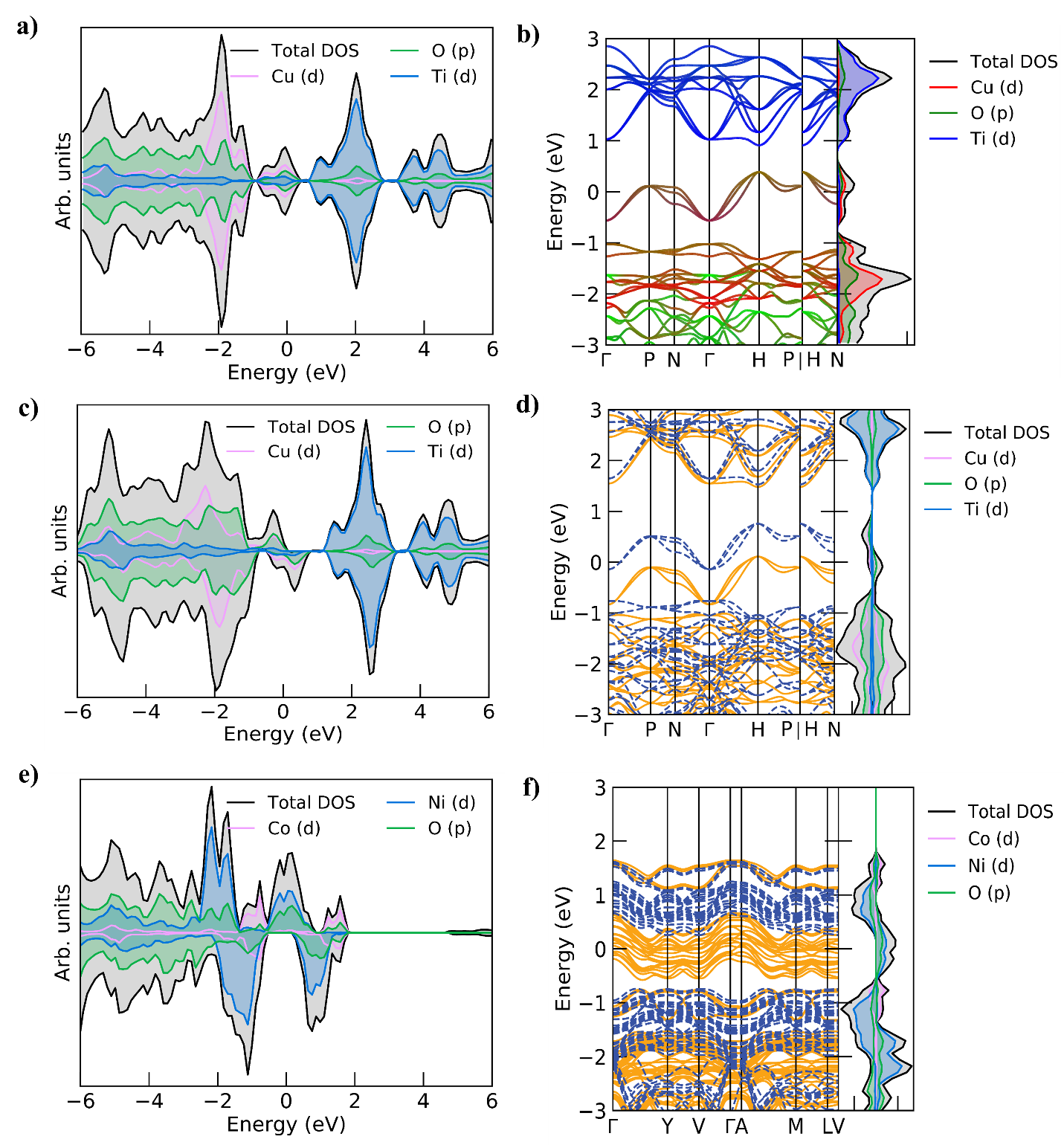


**Fig. S9** Density of states and energy band structure of spin-symmetric anti-ferromagnetic ordered CCTO of specifically Cu-d, O-s, and Ti-d (**a-b**); Density of states and energy band structure of spin-asymmetric CCTO of specifically Cu-d, O-s, and Ti-d (**c-d**); Density of states and energy band structure of NCAL of specifically Ni-d, Co-d, and O-s (**e-f**)


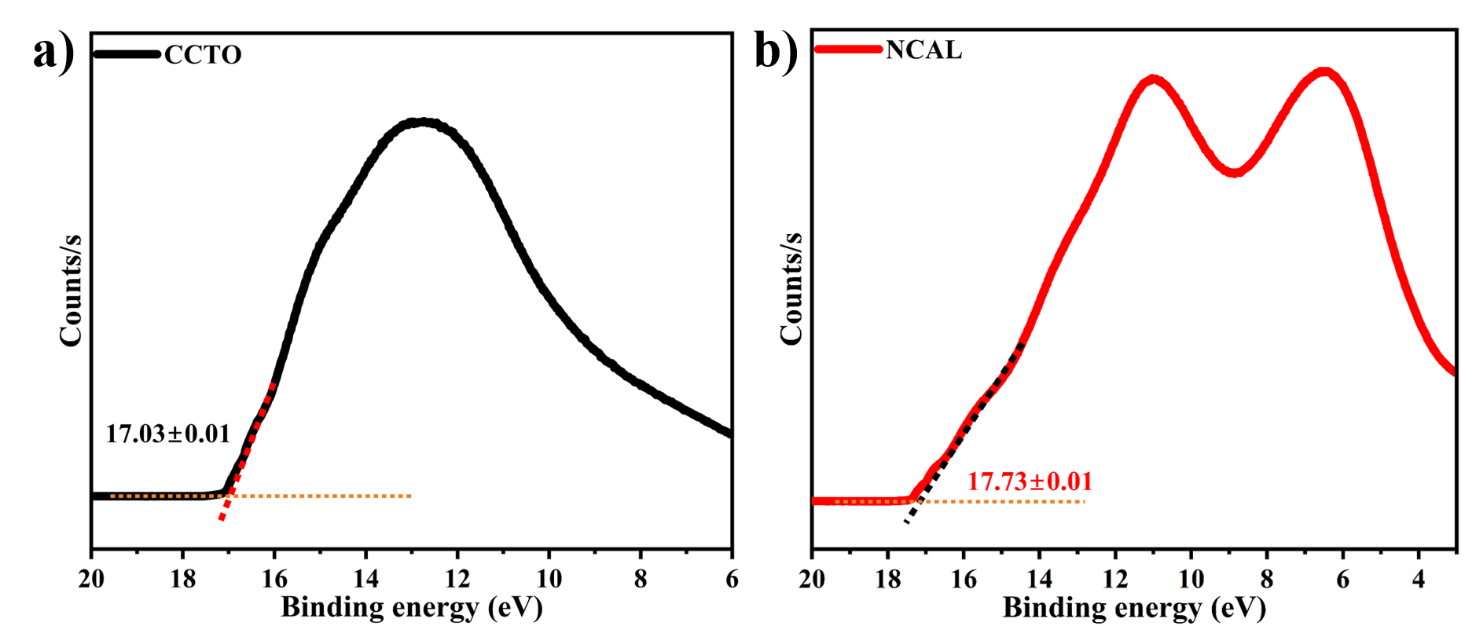


**Fig. S10** The cut-off results at higher binding energy of UPS of CCTO and NCAL (**a-b**)

Moreover, each single cell containing the CCTO-NCAL electrolyte and NCAL electrodes showed a high fuel conversion efficiency under three distinct current densities. At each current density, the outlet products were subjected to on-line GC analysis at 30-minute intervals (one GC run every 10 minutes for three measurements). For each current density, the H_2_O concentration was determined by taking the average value of the three runs, separated by 30 minutes. Fig. S11(a) illustrates the rise of concentration of H_2_O as applied current densities do, whereas the Faradaic efficiency decreases slightly. The Faradaic efficiency at a lower current density of 0.1 mA/cm^2^ is approximately 100% but slightly crosses over 100% because of the reduced water production brought about by the low applied current density; the GC-MS instrument's sensitivity is also connected to the slight cross over 100 % (shown in Fig. S11(a)). Thus, the very low H_2_O concentration produced can easily cause a possible deviation during the calibration and measurement of the GC–MS instrument. However, even at an applied current density of 0.2 mA cm^-2^, the H_2_O production and faradaic efficiency are still high, respectively.

The durability is an important parameter of the fuel cell device, therefore, the fuel cell device based on CCTO-NCAL electrolyte was operated under the applied constant current density of 110 mA cm^-2^ at 550 °C. It can be seen that fuel cell based on CCTO-NCAL electrolyte was operated for 21 hours as depicted in Fig. S11(b). Due to fluctuation of hydrogen fuel pressure led to the fluctuation of durability operation. Still, it is considered that fuel cell device based on CCTO-NCAL can be broadened and improved by employing the engineering support. In addition, it should be kept in mind that all experimental works are performed at the laboratory scale; therefore, many technical barriers exist that constrain and reduce the working voltage of the fuel cell device. Therefore, there is dire need to overcome the technical hurdles such as steel chamber of the sample holder from inside that resulted into increase of resistance and reduce conductivity, difference in cell geometry because of lack of engineering facilities that causes non-uniformity of the utilized NCAL-Ni electrode’ preparation and their employment in fuel cell device. All these technical hurdles and unavailability of engineering facilities limited the advancement of the fuel cell technology. Moreover, the mismatch of electrode and electrolyte in terms of thermal matching is a big hurdle; it is expected that higher is the thermal mismatch low will be the stable operation and non-sufficient output of fuel cell device and vice versa.


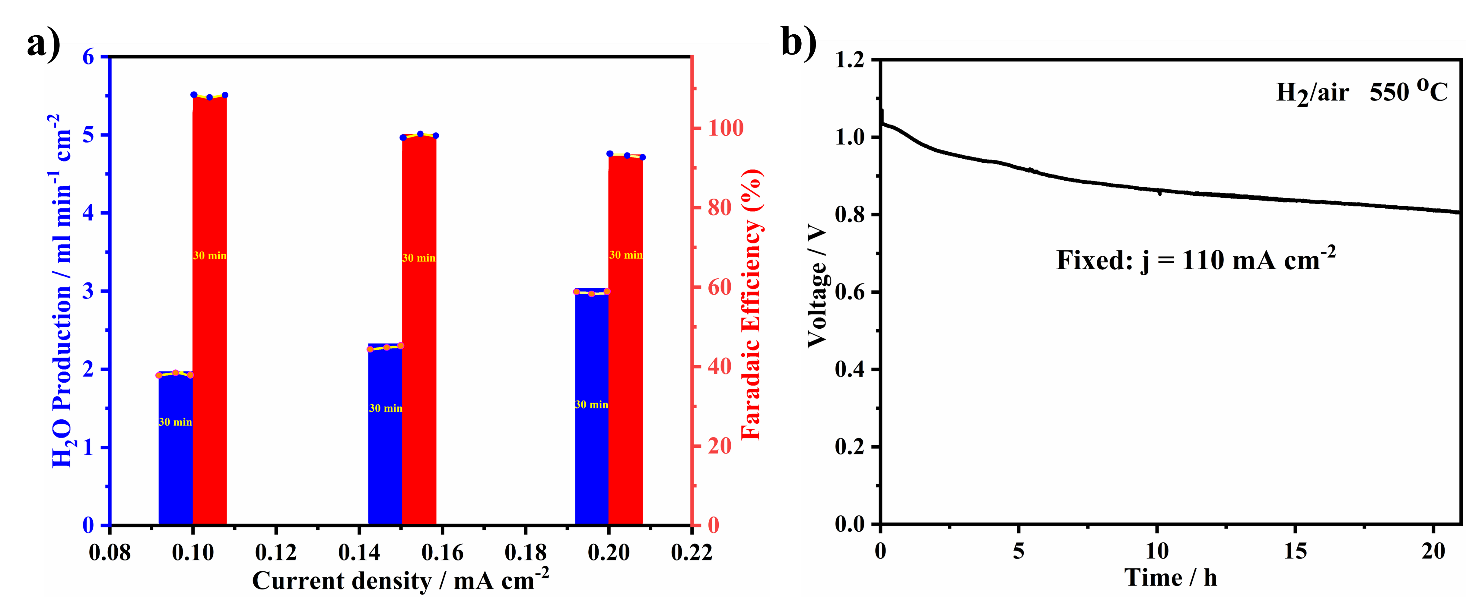


**Fig. S11** Performance of single cell based on comparison of Faradaic efficiency of single cell H_2_ conversion to H_2_O under varying current densities at 550 °C and H_2_O production using NCAL/CCTO-NCAL/NCAL. Three average values were used for the plot and the Faraday efficiency calculation took into account the average value of the three measurements (**a**) and Stability measurement of CCTO-NCAL SOFC at a fixed current density of 110 mA cm^-2^ at 550 °C for 21 h. The cell voltage started to deviates from the initial value for the prolonged time of over 21 h, which should be by limited by the used fuel cell testing fixture and NCAL electrode (**b**)
